# Synthesis and Characterization of All-Acrylic Tetrablock Copolymer Nanoparticles: Waterborne Thermoplastic Elastomers via One-Pot RAFT Aqueous Emulsion Polymerization

**DOI:** 10.1021/acs.chemmater.3c03115

**Published:** 2024-02-14

**Authors:** Oliver J. Deane, Pierre Mandrelier, Osama M. Musa, Mohammed Jamali, Lee A. Fielding, Steven P. Armes

**Affiliations:** † Department of Chemistry, University of Sheffield, Dainton Building, Brook Hill, Sheffield, South Yorkshire S3 7HF, U.K.; ‡ Ashland Specialty Ingredients, 1005 US 202/206, Bridgewater, New Jersey 08807, United States; § Department of Materials, School of Natural Sciences, The University of Manchester, Oxford Road, Manchester M13 9PL, U.K.; ∥ Henry Royce Institute, The University of Manchester, Oxford Road, Manchester M13 9PL, U.K.

## Abstract

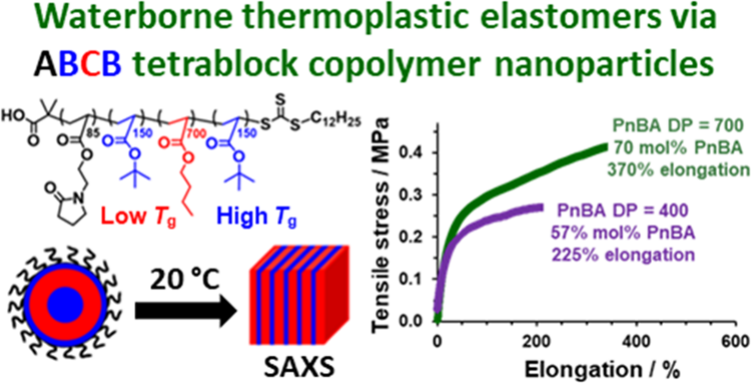

Reversible addition–fragmentation chain transfer (RAFT) aqueous emulsion polymerization is used to prepare well-defined ABCB tetrablock copolymer nanoparticles via sequential monomer addition at 30 °C. The A block comprises water-soluble poly(2-(*N*-acryloyloxy)ethyl pyrrolidone) (PNAEP), while the B and C blocks comprise poly(*t*-butyl acrylate) (PtBA) and poly(*n*-butyl acrylate) (PnBA), respectively. High conversions are achieved at each stage, and the final sterically stabilized spherical nanoparticles can be obtained at 20% w/w solids at pH 3 and at up to 40% w/w solids at pH 7. A relatively long PnBA block is targeted to ensure that the final tetrablock copolymer nanoparticles form highly transparent films on drying such aqueous dispersions at ambient temperature. The kinetics of polymerization and particle growth are studied using ^1^H nuclear magnetic resonance spectroscopy, dynamic light scattering, and transmission electron microscopy, while gel permeation chromatography analysis confirmed a high blocking efficiency for each stage of the polymerization. Differential scanning calorimetry and small-angle X-ray scattering studies confirm microphase separation between the hard PtBA and soft PnBA blocks, and preliminary mechanical property measurements indicate that such tetrablock copolymer films exhibit promising thermoplastic elastomeric behavior. Finally, it is emphasized that targeting an overall degree of polymerization of more than 1000 for such tetrablock copolymers mitigates the cost, color, and malodor conferred by the RAFT agent.

## Introduction

Block copolymers based on vinyl monomers are used for various commercial applications, ranging from thermoplastic elastomers^1^ to pressure-sensitive adhesives^2^ to lubricant additives,^3^ to dispersants for pigments and diesel soot.^4,5^ Traditionally, well-defined block copolymers have been prepared in organic solvents using living anionic polymerization.^6−9^ If nanoparticles were desired, they were typically obtained by a post-polymerization processing step such as a solvent switch or thin-film rehydration.^10,11^ However, such approaches typically only lead to relatively dilute dispersions, which have hitherto limited potential applications. In contrast, polymerization-induced self-assembly (PISA) can be used to prepare block copolymer nanoparticles directly in the form of concentrated colloidal dispersions.^12−17^

PISA syntheses of diblock copolymer nanoparticles in aqueous media involve reversible addition–fragmentation chain transfer (RAFT) dispersion or emulsion polymerization.^18−29^ However, there are relatively few examples of the synthesis of multiblock copolymer nanoparticles using this approach.^18−25^ In 2013, Gody et al. reported the one-pot multistep sequential RAFT *solution* polymerization of various acrylamides and acrylates to prepare a wide range of relatively well-defined multiblock copolymers (*M*_w_/*M*_n_ < 1.40) on a multigram scale.^18^ This pioneering study utilized a relatively low initiator concentration to minimize the formation of dead chains. Normally, this approach leads to slower polymerizations and/or lower final monomer conversions, but the relatively high propagation rate coefficients of acrylamides and acrylates enabled almost complete monomer conversion to be achieved for each block. However, only a relatively low degree of polymerization (DP) was targeted for each block to maximize RAFT control. Unfortunately, such multiblock copolymers are less likely to exhibit microphase separation in the solid state.^9,26,27^ Furthermore, targeting higher block DPs via RAFT solution polymerization would inevitably lead to significantly slower rates of polymerization and highly viscous copolymer solutions.

It is well-documented that anionic polymerization can be used to prepare well-defined ABA triblock copolymers, where the outer A blocks comprise polystyrene (PS) and the central B block is either polyisoprene (PI) or polybutadiene (PBD).^6^ Enthalpic incompatibility leads to microphase separation, with the PS blocks (*T*_g_ ∼100 °C) forming hard, glassy domains^28,29^ embedded within a soft, rubbery matrix formed by the low-*T*_g_ PI (or PBD) block.^9,30,31^ The PS domains act as physical cross-links and, if the soft central block is sufficiently long, a so-called synthetic rubber or thermoplastic elastomer is obtained. Such ABA triblock copolymers are highly flexible and extendable at ambient temperature: the soft block chains uncoil when the material is stretched, and the physical cross-links ensure full elastic recovery once the applied stress is removed.^32,33^ The microphase separation of block copolymers depends on the copolymer architecture, the relative block volume fractions, and a sufficiently high *χN* parameter, where χ is the Flory–Huggins interaction parameter and *N* is the mean DP.^27,34−36^ It is well known that high *N* values favor strong segregation, whereas no microphase separation (or only weak segregation) is observed for relatively low *N* values.^37^ In practice, strong segregation usually occurs if χ*N >* 10.^26^ The ultimate mechanical properties of meth(acrylate)-based thermoplastic elastomers are often relatively poor compared to the traditional styrene-diene-styrene-based thermoplastic elastomers.^38−41^ This is mainly because the former system requires a much higher minimum molecular weight between chain entanglements (*M*_e_), e.g., *M*_e_ = 28.0 kg mol^–1^ for poly(*n*-butyl acrylate) [PnBA]^41^ compared to *M*_e_ = 1.7 and 6.1 kg mol^–1^ for PBD and PI, respectively.^41,42^

In principle, RAFT aqueous emulsion polymerization offers an attractive route for the direct synthesis of high molecular weight multiblock copolymer nanoparticles in concentrated aqueous media.^20−22,43−46^ This approach involves polymerizing a water-immiscible monomer from one end of a RAFT agent-capped water-soluble precursor, which acts as a steric stabilizer.^12,47^ The growing hydrophobic block becomes insoluble at a relatively low critical DP, which leads to micellar nucleation. The unreacted monomer then diffuses into these nascent nanoparticles, which become monomer-swollen. The resulting high local monomer concentration leads to a significant increase in the rate of polymerization.^48−51^ This enables a relatively low initiator concentration to be employed, which maximizes the pseudo-living character of the copolymer chains. In principle, such formulations offer a convenient low-viscosity route to high-molecular-weight polymers. Nevertheless, there are surprisingly few literature reports of the synthesis of multiblock copolymer nanoparticles by RAFT aqueous emulsion polymerization.^21,22,52,53^

Wang et al. used this approach to prepare well-defined, high molecular weight polystyrene-based nanoparticles.^54^ The weak polyelectrolyte character of the poly(acrylic acid) (PAA) precursor meant that the initial solution pH strongly influenced the ensuing styrene polymerization. Thus, the RAFT emulsion polymerization of styrene was initially performed at low pH to ensure good RAFT control. After the onset of micellar nucleation, the solution pH was raised to confer electrosteric stabilization. This protocol afforded optimal RAFT control (*M*_n_ up to 544 kg mol^–1^; *M*_w_/*M*_n_ < 1.26). Subsequently, Luo et al. used this pH-switch method to prepare (PAA_27_-PS_5_)-PS_*x*_-PnBA_*y*_-PS_*x*_ tetrablock copolymer nanoparticles via sequential RAFT emulsion polymerization of styrene (for 70 min), nBA (for 50 min), and styrene (for 85 min) under monomer-starved conditions.^20^^1^H NMR studies confirmed that more than 90% conversion was achieved for each block, which reduced (but did not eliminate) the formation of gradient copolymers at each stage. A series of relatively well-defined tetrablock copolymers (*M*_w_/*M*_n_ = 1.41) were obtained when targeting an overall copolymer *M*_n_ of 86.1 kg mol^–1^, but significantly broader molecular weight distributions (*M*_w_/*M*_n_ = 1.63–3.19) were observed when targeting higher molecular weights (*M*_n_ = 112–338 kg mol^–1^) and PnBA mass fractions of more than 60%. These (PAA_27_-PS_5_)-PS_*x*_-PnBA_*y*_-PS_*x*_ tetrablock copolymers were then isolated and solubilized as 10% w/w copolymer solutions in THF. Subsequently, films were obtained by (i) casting these solutions at room temperature, (ii) allowing solvent evaporation to occur for 72 h, and (iii) drying the resulting films to constant weight in a vacuum oven for 24 h at 120 °C (i.e., above the *T*_g_ of the PS). The mechanical properties of this series of thermoplastic elastomer films were impressive: tensile strengths of ∼10 MPa and elongation at break values of 500% were obtained for PS mass contents of 40–50%. However, the film formation protocol is not attractive from an industrial perspective: it would be far more desirable to obtain thermoplastic elastomer films directly from waterborne multiblock copolymer nanoparticles without utilizing any organic solvent. Similarly, Li et al. reported the synthesis of ABCBA pentablock copolymer nanoparticles via RAFT aqueous emulsion polymerization using (semi)fluorinated vinyl monomers. In this case, transparent thermoplastic elastomeric films with impressive mechanical properties could be obtained but only after thermal annealing at 120 °C.^55^

More recently, a PAA_27_-PS_5_ precursor was employed by Guimarães et al. to prepare multiblock copolymers comprising four or more blocks via RAFT aqueous emulsion copolymerization.^21^ A “nonablock” PS homopolymer was prepared with at least 92% conversion being achieved within 3 h for each PS block (total reaction time = 9 × 3 = 27 h). However, after the sixth PS block, the molecular weight distributions became broader (*M*_w_/*M*_n_ = 1.4–1.7), indicating a gradual loss of RAFT control. Transmission electron microscopy (TEM) and gel permeation chromatography (GPC) studies confirmed that the mean contour length of the copolymer chains was comparable to the nanoparticle radius. In principle, this means that one end of the copolymer chain is located at the nanoparticle surface while the other is located within the nanoparticle cores. In the same study, the RAFT aqueous emulsion homopolymerization of nBA was also attempted. However, only a relatively low monomer conversion was obtained, despite employing a higher initiator concentration and longer polymerization times and adjusting the solution pH. Indeed, high nBA conversions could only be achieved if 10% styrene was added to the nBA monomer feed, with this statistical copolymerization being performed over 18 h at 75 °C. This approach enabled the preparation of a P(AA_27_-PS_5_)-PS_200_-P(nBA-*stat*-S)_200_-PS_200_-P(nBA-*stat*-S)_200_-PS_200_ hexablock copolymer, but GPC analysis indicated that the molecular weight distribution became significantly broader (*M*_w_/*M*_n_ > 1.50) after the third block. TEM studies of such copolymer nanoparticles were not possible owing to their film formation during sample grid preparation. The nBA was then replaced with *n*-butyl methacrylate (BMA). TEM studies confirmed that the resulting hexablock copolymer underwent microphase separation, which suggested the formation of a well-defined multilayered onion-like structure within the nanoparticles. However, statistical copolymerization of styrene within the P(S-*stat*-BMA)_200_ blocks inevitably leads to a *T*_g_ that is too high for film formation at ambient temperature, while the relatively long polymerization time (18 h) required to generate the P(S-*stat*-BMA)_200_ block is not desirable for industrial scale-up.

**Scheme 1:**
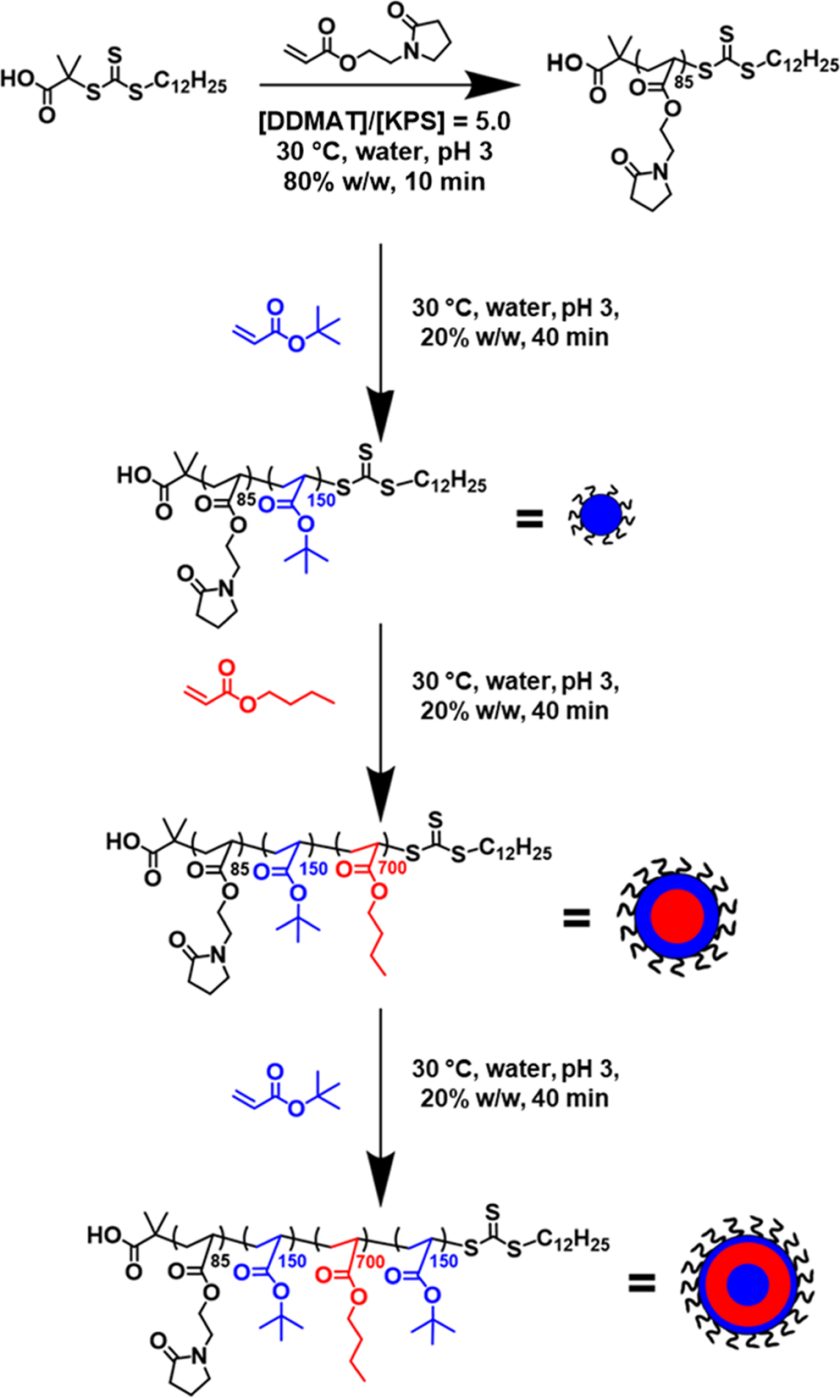
Synthesis of PNAEP_85_-PtBA_150_-PnBA_700_-PtBA_150_ Tetrablock Copolymer Nanoparticles via Initial RAFT Aqueous Solution Polymerization, Followed by Three Successive RAFT Aqueous Emulsion Polymerizations Conducted at 30 °C Initiator was added with each monomer addition using a [potassium persulfate]/[ascorbic acid] ([KPS]/[AsAc]) molar ratio of 1.0 and [trithiocarbonate]/[KPS] molar ratio of 5.0 for each tBA polymerization and 10.0 for the NAEP and nBA polymerizations. Each polymerization was allowed to proceed at pH 3 until at least 95% conversion had been achieved prior to addition of the next monomer. Water was also added at each stage to maintain an overall copolymer concentration of 20% w/w solids. See Scheme S1 for the analogous synthesis route conducted at pH 7, which leads to anionic carboxylate groups on the stabilizer chains.

Herein we report the efficient synthesis of new all-acrylic thermoplastic elastomer multiblock copolymer nanoparticles via sequential RAFT aqueous emulsion copolymerization of nBA and *t*-butyl acrylate (tBA) using a highly hydrophilic poly(2-(*N*-acryloyloxy)ethyl pyrrolidone) (PNAEP) block^56,57^ as a steric stabilizer (see Scheme 1). It is well known that the propagation rate constants of acrylics are much higher than that of the corresponding methacrylates (or styrene).^58,59^ Thus high monomer conversions should be achieved for each block within relatively short reaction times. Moreover, despite tBA and nBA being structural isomers, they are distinguishable by ^1^H NMR spectroscopy, so this technique can be used to monitor the polymerization kinetics. Furthermore, the *T*_g_ of the PtBA block is sufficiently high (up to 50 °C)^60^ compared to that of PnBA (*T*_g_ = −54 °C)^61−63^ that thermoplastic elastomer films were anticipated when drying such PNAEP-PtBA-PnBA-PtBA tetrablock copolymer dispersions at ambient temperature. Finally, the all-acrylic nature of such nanoparticles should ensure the formation of highly transparent films.^64^

## Experimental Section

### Materials

*N*-(2-(Acryloyloxy)ethyl)pyrrolidone (NAEP; 95% purity) was provided by Ashland Specialty Ingredients (Cherry Hill, NJ) and was further purified by dilution with chloroform followed by sequential washes with 5% Na_2_CO_3_ solution, saturated NaCl solution, and finally, deionized water. Repeated washes with water were conducted until the NAEP solution was neutralized, prior to drying over anhydrous MgSO_4_. All chemicals used for NAEP purification were purchased from Sigma-Aldrich UK and were used as received. Potassium persulfate (KPS), ascorbic acid (AsAc), tetramethylethylenediamine (TMEDA), 2-(dodecylthiocarbonothioylthio)-2-methylpropionic acid (DDMAT; 98%), *n*-butyl acrylate (nBA), *tert*-butyl acrylate (tBA), hydrochloric acid (1.0 M), and sodium hydroxide (1.0 M) were purchased from Sigma-Aldrich (Dorset, U.K.). CD_3_OD was purchased from Goss Scientific Instruments Ltd. (Cheshire, UK). All other solvents were purchased from Fisher Scientific (Loughborough, U.K.) and were used as received. Deionized water was used for all experiments, and the solution pH was adjusted using either 0.1 M HCl or 0.1 M NaOH.

#### One-Pot Synthesis of PNAEP_85_-PtBA_150_-PnBA_*x*_-PtBA_150_ Tetrablock Copolymer Nanoparticles via Sequential RAFT Emulsion Polymerization at pH 3

A typical protocol used for the one-pot synthesis of PNAEP_85_-P*t*BA_150_-P*n*BA_700_-P*t*BA_150_ tetrablock copolymer nanoparticles at 20% w/w solids was conducted as follows. DDMAT RAFT agent (8.0 mg, 21.83 μmol) was added to NAEP (0.300 g, 1.64 mmol; target PNAEP DP = 75) and KPS (0.80 mg, 4.37 μmol; [DDMAT]/[KPS] molar ratio = 5.0) in a 28 mL glass vial charged with a magnetic flea (reaction solution 1). This vial was then placed in an ice bath, and nitrogen was passed over the top of the solution for 30 min. Then, the vial was immersed in an oil bath set at 30 °C. AsAc (1.20 mg, 4.37 μmol; [DDMAT]/[ASAC] molar ratio = 5.0; [KPS]/[ASAC] molar ratio = 1.0) and deionized water adjusted to pH 3 using HCl (75.5 mg; pH 3; final solids concentration = 80% w/w) were combined and degassed before being added via a degassed syringe/needle to the glass vial containing reaction solution 1 under a nitrogen atmosphere. The ensuing NAEP polymerization was allowed to proceed for 10 min prior to dilution of the viscous aqueous reaction solution via addition of degassed deionized water (1.31 g; pH 3; final target solids concentration = 20% w/w). The resulting reaction solution was then stirred magnetically for 2 min to ensure dissolution of the PNAEP homopolymer. A degassed syringe/needle was used to extract an aliquot for ^1^H NMR spectroscopy analysis. The reduction in the monomer vinyl signals at 5.9 and 6.4 ppm relative to the integrated four ethyl protons at 3.4–3.8 ppm assigned to PNAEP indicated an NAEP conversion of 98%. The mean DP of this PNAEP precursor was calculated to be 85, as judged by ^1^H NMR studies in CD_3_OD [the integrated signal at 3.4–3.8 ppm (m, 4H) was compared to that at 0.86–0.96 ppm (t, 3H) assigned to the methyl end group of the RAFT agent]. DMF GPC analysis indicated an *M*_n_ of 15.5 kg mol^–1^ and an *M*_w_/*M*_n_ of 1.15 (expressed relative to a series of poly(methyl methacrylate) calibration standards). To generate the first PtBA block, degassed tBA (0.370 g, 2.92 mmol; PtBA target DP = 150) was added to the reaction solution. KPS (0.53 mg, 1.94 μmol; [PNAEP_85_]/[KPS] molar ratio = 5.0) and AsAc (0.34 mg, 1.94 μmol; [PNAEP_85_]/[AsAc] molar ratio = 5.0; [KPS]/[ASAC] molar ratio = 1.0) were added to the reaction mixture as dilute aqueous solutions (0.13 mM and 0.08 mM, respectively) using degassed syringe/needles. The tBA polymerization was allowed to proceed for 30 min at 30 °C prior to dilution of the viscous aqueous reaction solution via addition of degassed deionized water (1.75 g; pH 7; final target solids concentration = 20% w/w). ^1^H NMR spectroscopy studies indicated a final tBA conversion of 98%. The mean DP of the PtBA block was calculated to be 150, as judged by ^1^H NMR spectroscopy analysis in CD_3_OD [the integrated signal at 1.5 ppm (1350H) was compared to that assigned to two oxymethylene protons assigned to PNAEP_85_ at 2.1–2.2 ppm (m, 170H), see Figure 3]. To generate the PnBA block, degassed nBA (1.75 g, 13.62 mmol; PnBA target DP = 700) was added to the reaction solution. KPS (1.05 mg, 3.90 μmol; [PNAEP_85_-PtBA_150_]/[KPS] molar ratio = 10.0) and ASAC (0.69 mg, 3.90 μmol; [PNAEP_85_-PtBA_150_]/[ASAC] molar ratio = 10.0; [KPS]/[ASAC] molar ratio = 1.0) were added to the reaction mixture as dilute aqueous solutions (0.13 and 0.08 mM, respectively) using degassed syringe/needles. The nBA polymerization was allowed to proceed for 40 min at 30 °C prior to dilution of the viscous aqueous reaction solution via addition of degassed deionized water (0.75 g; pH 7; final target solids concentration = 20% w/w). ^1^H NMR studies indicated a final nBA conversion of 97%. The mean DP of the PtBA block was calculated to be 700, as judged by ^1^H NMR analysis in CD_3_OD [the integrated signal at 0.95 ppm (2100 H) was compared to that assigned to four ethyl protons assigned to PNAEP_85_ at 3.5–3.6 ppm (m, 340H)] (see Figure 3). DMF GPC analysis indicated an *M*_n_ of 114.6 kg mol^–1^ and an *M*_w_/*M*_n_ of 1.54. To generate the second PtBA block, degassed tBA (0.370 g, 2.92 mmol; PtBA target DP = 150) was added to the reaction solution. KPS (0.53 mg, 1.94 μmol; [PNAEP_85_–PtBA_150_-PnBA_700_]/[KPS] molar ratio = 5.0) and ASAC (0.34 mg, 1.94 μmol; [PNAEP_85_-PtBA_150_-PnBA_700_]/[ASAC] molar ratio = 5.0; [KPS]/[ASAC] molar ratio = 1.0) were added to the reaction mixture as dilute aqueous solutions (0.13 mM and 0.08 mM, respectively) using degassed syringe/needles. The tBA polymerization was allowed to proceed for 30 min at 30 °C before being quenched by exposing the reaction mixture to air and immersing the glass vial into an ice bath. ^1^H NMR studies indicated a final tBA conversion of 99%. The mean DP of the PtBA block was calculated to be 150, as judged by ^1^H NMR analysis in CD_3_OD [the integrated signal at 1.5 ppm (total = 2700H, subtract 1350H from the original PtBA_150_ block indicates 1350H for the second PtBA block) was compared with that assigned to four ethyl protons assigned to PNAEP_85_ at 3.5–3.6 ppm (m, 340H)] (see Figure 3). DMF GPC analysis indicated an *M*_n_ of 131.5 kg mol^–1^ and an *M*_w_/*M*_n_ of 1.59 when calibrated against a series of poly(methyl methacrylate) standards. Other tetrablock copolymer compositions were targeted by adjusting the [nBA]/[PNAEP_85_-PtBA_150_] molar ratio accordingly.

#### One-Pot Synthesis of PNAEP_85_-PtBA_150_-PnBA_*x*_-PtBA_150_ Tetrablock Copolymer Nanoparticles via Sequential RAFT Emulsion Polymerization at pH 7

A typical protocol used for the one-pot synthesis of PNAEP_85_-P*t*BA_150_-P*n*BA_700_-P*t*BA_150_ tetrablock copolymer nanoparticles at 20% w/w solids was conducted as follows. DDMAT RAFT agent (8.0 mg, 21.83 μmol) was added to NAEP (0.300 g, 1.64 mmol; target PNAEP DP = 75) and KPS (0.80 mg, 4.37 μmol; [DDMAT]/[KPS] molar ratio = 5.0) in a 28 mL glass vial charged with a magnetic flea (reaction solution 1). This vial was then placed in an ice bath, and nitrogen was passed over the top of the solution for 30 min. Then, the vial was immersed in an oil bath set at 30 °C. TMEDA (1.20 mg, 4.37 μmol; [DDMAT]/[TMEDA] molar ratio = 5.0; [KPS]/[TMEDA] molar ratio = 1.0) and deionized water adjusted to pH 7 using NaOH (75.5 mg; pH 7; final solids concentration = 80% w/w) were combined and degassed before being added via a degassed syringe/needle to the glass vial containing reaction solution 1 under a nitrogen atmosphere. The ensuing NAEP polymerization was allowed to proceed for 10 min prior to dilution of the viscous aqueous reaction solution via addition of degassed deionized water (1.31 g; pH 7; final target solids concentration = 40% w/w). The resulting reaction solution was then stirred magnetically for 2 min to ensure dissolution of the PNAEP homopolymer. A degassed syringe/needle was used to extract an aliquot for ^1^H NMR spectroscopy analysis. The reduction in the monomer vinyl signals at 5.9 and 6.4 ppm relative to the integrated four ethyl protons at 3.4–3.8 ppm assigned to PNAEP indicated an NAEP conversion of 98%. The mean DP of this PNAEP precursor was calculated to be 85, as judged by ^1^H NMR studies in CD_3_OD [the integrated signal at 3.4–3.8 ppm (m, 4H) was compared to that at 0.86–0.96 ppm (t, 3H) assigned to the methyl end group of the RAFT agent]. To generate the first PtBA block, degassed tBA (0.370 g, 2.92 mmol; PtBA target DP = 150) was added to the reaction solution. KPS (0.53 mg, 1.94 μmol; [PNAEP_85_]/[KPS] molar ratio = 5.0) and TMEDA (0.34 mg, 1.94 μmol; [PNAEP_85_]/[TMEDA] molar ratio = 5.0; [KPS]/[TMEDA] molar ratio = 1.0) were added to the reaction mixture as dilute aqueous solutions (0.13 mM and 0.08 mM, respectively) using degassed syringe/needles. The tBA polymerization was allowed to proceed for 30 min at 30 °C prior to dilution of the viscous aqueous reaction solution via addition of degassed deionized water (1.75 g; pH 7; final target solids concentration = 40% w/w). ^1^H NMR spectroscopy studies indicated a final tBA conversion of 98%. The mean DP of the PtBA block was calculated to be 150, as judged by ^1^H NMR spectroscopy analysis in CD_3_OD. To generate the PnBA block, degassed nBA (1.75 g, 13.62 mmol; PnBA target DP = 700) was added to the reaction solution. KPS (1.05 mg, 3.90 μmol; [PNAEP_85_-PtBA_150_]/[KPS] molar ratio = 10.0) and TMEDA (0.69 mg, 3.90 μmol; [PNAEP_85_-PtBA_150_]/[TMEDA] molar ratio = 10.0; [KPS]/[TMEDA] molar ratio = 1.0) were added to the reaction mixture as dilute aqueous solutions (0.13 and 0.08 mM, respectively) using degassed syringe/needles. The nBA polymerization was allowed to proceed for 40 min at 30 °C prior to dilution of the viscous aqueous reaction solution via addition of degassed deionized water (0.75 g; pH 7; final target solids concentration = 40% w/w). ^1^H NMR studies indicated a final nBA conversion of 96%. The mean DP of the PtBA block was calculated to be 700, as judged by ^1^H NMR analysis in CD_3_OD. To generate the second PtBA block, degassed tBA (0.370 g, 2.92 mmol; PtBA target DP = 150) was added to the reaction solution. KPS (0.53 mg, 1.94 μmol; [PNAEP_85_-PtBA_150_-PnBA_700_]/[KPS] molar ratio = 5.0) and TMEDA (0.34 mg, 1.94 μmol; [PNAEP_85_-PtBA_150_-PnBA_700_]/[TMEDA] molar ratio = 5.0; [KPS]/[TMEDA] molar ratio = 1.0) were added to the reaction mixture as dilute aqueous solutions (0.13 and 0.08 mM, respectively) using degassed syringe/needles. The tBA polymerization was allowed to proceed for 30 min at 30 °C before being quenched by exposing the reaction mixture to air and immersing the glass vial into an ice bath. ^1^H NMR studies indicated a final tBA conversion of 99%. The mean DP of the PtBA block was calculated to be 150, as judged by ^1^H NMR analysis in CD_3_OD. Other tetrablock copolymer compositions were targeted by adjusting the [nBA]/[PNAEP_85_–PtBA_150_] molar ratio accordingly.

### Copolymer Characterization

#### ^1^H NMR Spectroscopy

Spectra were recorded in CD_3_OD using a 400 MHz Bruker AVANCE-400 spectrometer, with 64 scans being averaged per spectrum.

#### Gel Permeation Chromatography

Copolymer molecular weights and dispersities were determined using an Agilent 1260 Infinity GPC system equipped with a refractive index detector and a UV–visible detector. Two Agilent PLgel 5 μm Mixed-C columns and a guard column were connected in series and maintained at 60 °C. HPLC-grade DMF containing 10 mM LiBr was used as the eluent, and the flow rate was set at 1.0 mL min^–1^. A refractive index detector was used to calculate molecular weights and dispersities using a series of 10 near-monodisperse poly(methyl methacrylate) calibration standards (with *M*_n_ values ranging from 370 to 2,520,000 g mol^–1^).

#### Transmission Electron Microscopy

As-prepared copolymer dispersions were diluted to 0.1% w/w at 20 °C using, where appropriate, dilute aqueous HCl (pH 3) or NaOH (pH 7). Copper/palladium TEM grids (Agar Scientific, U.K.) were coated in-house to produce thin films of amorphous carbon. These grids were then treated with a plasma glow discharge for 30 s to create a hydrophilic surface. One droplet of an aqueous copolymer dispersion (20 μL; 0.1% w/w) was placed on a freshly treated grid for 1 min and then blotted with a filter paper to remove excess solution. To stain the deposited nanoparticles, an aqueous solution of uranyl formate (10 μL; 0.75% w/w) was placed on the sample-loaded grid via micropipette for 45 s and then carefully blotted to remove excess stain. Each grid was then dried using a vacuum hose. Imaging was performed using a Philips CM100 instrument operating at 100 kV and equipped with a Gatan 1k CCD camera.

#### Dynamic Light Scattering

Measurements were conducted at 25 °C using a Malvern Instruments Zetasizer Nano ZS instrument equipped with a 4 mW He–Ne laser (λ = 633 nm) and an avalanche photodiode detector. Scattered light was detected at 173°, and copolymer dispersions were diluted to 0.10% w/w prior to analysis. Measurements were averaged over three runs, and z-average hydrodynamic diameters were calculated using the Stokes–Einstein equation.

#### Differential Scanning Calorimetry

DSC studies were performed on (co)polymer powders or films using a TA Instruments Discovery DSC instrument equipped with Tzero low-mass aluminum pans and hermetically sealed lids. Each copolymer was equilibrated above its *T*_g_ for 10 min before performing two consecutive thermal cycles at a heating/cooling rate of 10 °C min^–1^. Two cycles were performed to eliminate any thermal history.

#### Copolymer Film Preparation

The as-prepared 20–40% w/w copolymer dispersions were allowed to dry on PTFE sheets in a 4 cm × 2 cm area at 20 °C in a fume cupboard for 24 h. The resulting films were then peeled off to produce free-standing films. The copolymer film thickness could be varied between 50 and 200 μm (±10 μm) by drying larger volumes of the 40% w/w dispersion (1.0 ± 0.5–5.0 ± 0.5 g, respectively).

#### Mechanical Properties

Preliminary tensile tests were performed by stretching copolymer films by hand, with digital photographs being recorded in their original relaxed state and at their maximum elongation prior to film rupture. Changes in film length were determined using a graduated ruler. After the stretched films were released, digital photographs were recorded to demonstrate complete contraction to their original dimensions.

#### Uniaxial Tensile Strength

Tensile performance of the tetrablock films was measured using a Static Testing Instron 3344L3927 fitted with a ±10 N static rating load cell. Latex films were prepared for tensile measurements by drop casting 10 g of the 20% w/w copolymer dispersions onto PTFE sheets (140 mm × 100 mm), which were subsequently left to dry at ambient conditions in a fume hood. After 7 days, the tetrablock films were peeled off the PTFE sheet and inverted to dry for the same period of time. A microtome blade was used to cut the films (0.5 mm thick) into rectangles (40 mm × 10 mm), which were loaded into paper frames to hold the film in place before clamping to the instrument. The paper frame was cut, and strain was applied to the films at a jog rate of 10 mm min^–1^. Young’s moduli were calculated from the gradient of the obtained tensile stress–strain curves in the initial liner region. Toughness was calculated by integrating the area under the stress–strain curves. Each measurement described above was conducted in triplicate.

#### Small-Angle X-ray Scattering Studies

SAXS studies were conducted on 150 ± 10 μm PNAEP_85_-PtBA_150_-PnBA_400_-PtBA_150_ and PNAEP_85_-PtBA_150_-PnBA_700_-PtBA_150_ copolymer films using a Xeuss 2.0 (Xenocs) SAXS instrument equipped with a FOX 3D multilayered X-ray mirror, two sets of scatterless slits for collimation, a hybrid pixel area detector (Pilatus 1M, Dectris), and a liquid gallium MetalJet X-ray source (Excillum, λ = 1.34 Å). SAXS patterns were recorded at a sample-to-detector distance of approximately 1.20 m (calibrated using a silver behenate standard). 2D SAXS patterns were reduced to 1D plots by azimuthal integration using the Foxtrot software package.

SAXS patterns of 1.0% w/w aqueous tetrablock copolymer dispersions were collected at Diamond Light Source (station I22, Didcot, UK) using monochromatic X-ray radiation (wavelength, λ = 0.124 nm, with *q* ranging from 0.015 to 1.3 nm^–1^, where *q* = 4π sin θ/λ is the length of the scattering vector and θ is one-half of the scattering angle) and a 2D Pilatus 2 M pixel detector (Dectris, Switzerland). Glass capillaries of 2.0 mm diameter were used as a sample holder. SAXS data were reduced (integration, normalization, and absolute intensity calibration using a SAXS pattern recorded for deionized water, assuming that the differential scattering cross section of water is 0.0162 cm^–1^) using Dawn software supplied by Diamond Light Source.^65^

## Results and Discussion

### Kinetic Studies During the One-Pot Synthesis of PNAEP_85_-PtBA_150_-PnBA_*x*_-PtBA_150_ Tetrablock Copolymer Nanoparticles via Sequential RAFT Emulsion Polymerization

The synthesis of block copolymer nanoparticles by RAFT aqueous emulsion polymerization typically involves using a water-soluble precursor that acts as the steric stabilizer for the growing nanoparticles.^51^ This precursor is often prepared by RAFT solution polymerization in a suitable organic solvent. Such polymerizations are usually terminated at intermediate conversion to avoid monomer-starved conditions, which can otherwise lead to premature loss of RAFT chain ends.^66^ However, robust one-pot protocols have now been developed in which high monomer conversion (>95%) is achieved for the soluble precursor, which is then chain-extended immediately without any purification.^67,68^ Accordingly, the synthesis of three aqueous dispersions of PNAEP_85_-PtBA_150_-PnBA_*x*_-PtBA_150_ (*x* = 200, 400, or 700) tetrablock copolymer nanoparticles was conducted via initial RAFT aqueous solution polymerization of NAEP and subsequent RAFT aqueous emulsion polymerization of tBA, nBA, and tBA, in turn, using a one-pot protocol (see Scheme 1).

**Figure 1:**
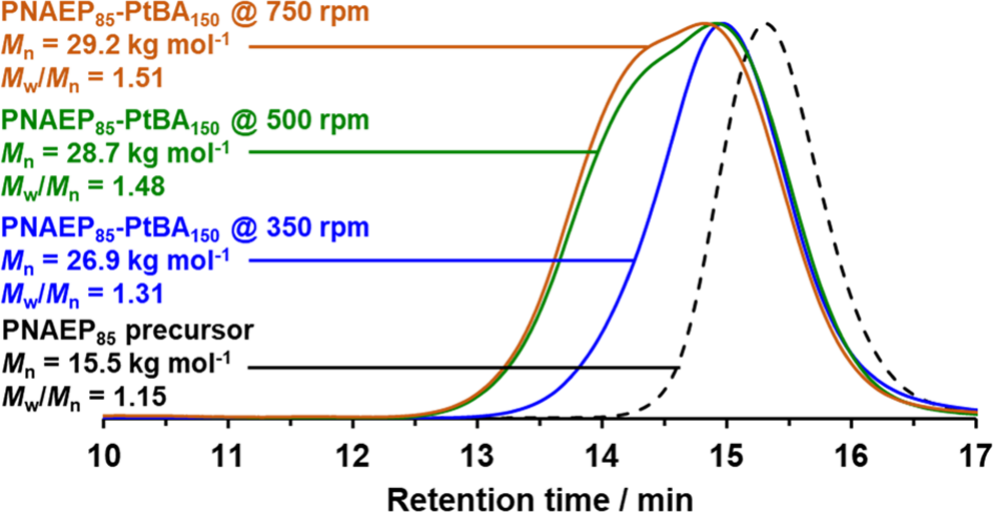
DMF GPC curves recorded for PNAEP_85_-PtBA_150_ diblock copolymers prepared using three different stirring rates (350, 500, or 750 rpm). Conducting the RAFT emulsion polymerization of tBA at pH 3 using lower stirring rates resulted in substantially incomplete monomer conversions. GPC data are expressed relative to a series of near-monodisperse poly(methyl methacrylate) calibration standards using a refractive index detector.

First, a water-soluble PNAEP_85_ precursor was prepared via RAFT aqueous solution polymerization of NAEP (Scheme 1). ^1^H NMR spectroscopy studies indicated that 98% NAEP conversion was achieved within 10 min. This relatively short time scale prevents premature loss of RAFT chain ends owing to hydrolysis or other side reactions.^69,70^ This PNAEP_85_ block was then chain-extended via RAFT aqueous emulsion polymerization of tBA with the same low-temperature redox initiator at 30 °C using a [PNAEP_85_]/[KPS] molar ratio of 5.0, see Scheme 1. However, further initiator had to be added to ensure that a high monomer conversion was achieved; otherwise, only 74% tBA conversion was obtained after 1 h at 30 °C (with no further increase in conversion being observed after 17 h at this temperature). DMF GPC studies performed on PNAEP_85_-PtBA_150_ diblock copolymers prepared at stirring rates of 350, 500, or 750 rpm indicated that the molecular weight distribution was rather sensitive to this parameter (see Figure 1).

Interestingly, the *slowest* rate of stirring produced the narrowest molecular weight distribution (*M*_w_/*M*_n_ = 1.31); stirring rates of either 500 or 750 rpm produced significantly higher *M*_w_/*M*_n_ values of 1.48 or 1.51, respectively. However, attempting to perform the polymerization at even slower stirring rates resulted in incomplete conversion (e.g., only 87% tBA conversion was achieved at 300 rpm). Thus, a stirring rate of 350 rpm was selected for all subsequent tetrablock copolymer syntheses. These empirical observations are consistent with similar experiments conducted by Boissé et al., who reported that increasing the stirring rate from 100 to 750 rpm led to faster reaction rates and progressively broader molecular weight distributions (e.g., *M*_w_/*M*_n_ values increased from 1.52 to 2.31) during the RAFT aqueous emulsion polymerization of styrene.^71^ Presumably, faster stirring leads to smaller emulsion droplets, which leads to more efficient mass transport of the water-immiscible tBA monomer into the growing nanoparticles. Indeed, Zetterlund and co-workers recently published a series of papers investigating the importance of mass transport of monomer during RAFT aqueous emulsion polymerization.^21,22,72,73^ In particular, Richardson et al. reported that the diffusion-limited mass transport of BMA from monomer droplets into the growing nanoparticle cores combined with the relatively high local concentration of RAFT CTA chain ends significantly lowers the effective BMA/CTA molar ratio.^72^ This reduces the number of monomer units added to each propagating chain per activation/deactivation cycle and hence leads to relatively narrow molecular weight distributions. However, the effect of varying the stirring rate was not examined. The GPC curves shown in Figure 1 suggest that this can be an important parameter for RAFT aqueous emulsion polymerization. This is consistent with the PISA literature for such heterogeneous formulations.^52,74^

**Figure 2:**
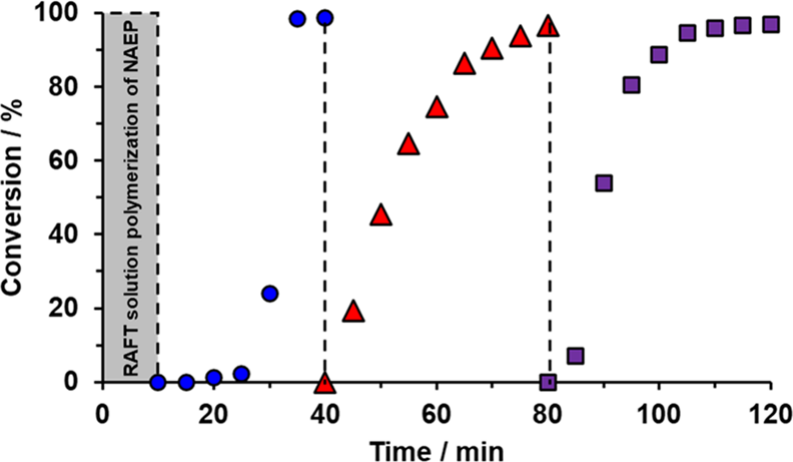
Conversion vs time curves determined by ^1^H NMR spectroscopy during the synthesis of PNAEP_85_-PtBA_150_-PnBA_700_-PtBA_150_ tetrablock copolymer nanoparticles at 30 °C via one-pot sequential RAFT aqueous emulsion copolymerization of tBA (blue circles), nBA (red triangles), and tBA (purple squares) using a PNAEP_85_ precursor prepared *in situ* within 10 min by RAFT aqueous solution polymerization of NAEP at 80% w/w solids (see gray region; kinetic data not shown). Target DPs were 150, 700, and 150 for the first PtBA block, the central PnBA block, and the second PtBA block, respectively. A [trithiocarbonate]/KPS molar ratio of 5.0 was used to prepare the two tBA blocks, whereas a [trithiocarbonate]/KPS molar ratio of 10.0 was employed for the polymerization of nBA and NAEP. Vertical dashed lines indicate injection times for tBA, nBA, and tBA during this aqueous PISA synthesis, which was conducted at pH 3 while targeting 20% w/w solids.

^1^H NMR spectroscopy was used to study the kinetics of the RAFT aqueous emulsion polymerization of tBA (target DP = 150) at 30 °C using a PNAEP_85_ precursor that was prepared in situ, see Figure 2. Periodic sampling of the reaction mixture involved dilution of each aliquot using CDCl_3_, which was then dried using MgSO_4_. ^1^H NMR studies indicated an induction period of approximately 5 min (see blue circles in Figure 2). After 15 min, the tBA conversion was only 2.2%. This was attributed to the relatively low concentration of this water-immiscible monomer within the aqueous continuous phase. Thereafter, micellar nucleation produces nascent nanoparticles, which results in 24% tBA conversion within 20 min (i.e., just 5 min after the onset of nucleation). After a further 10 min, 99% tBA conversion was achieved. Thus, an overall reaction time of 40 min—including the time required for the RAFT solution polymerization of NAEP—was required to produce the initial PNAEP_85_-PtBA_150_ diblock copolymer nanoparticles, see Figure 2.

**Figure 3:**
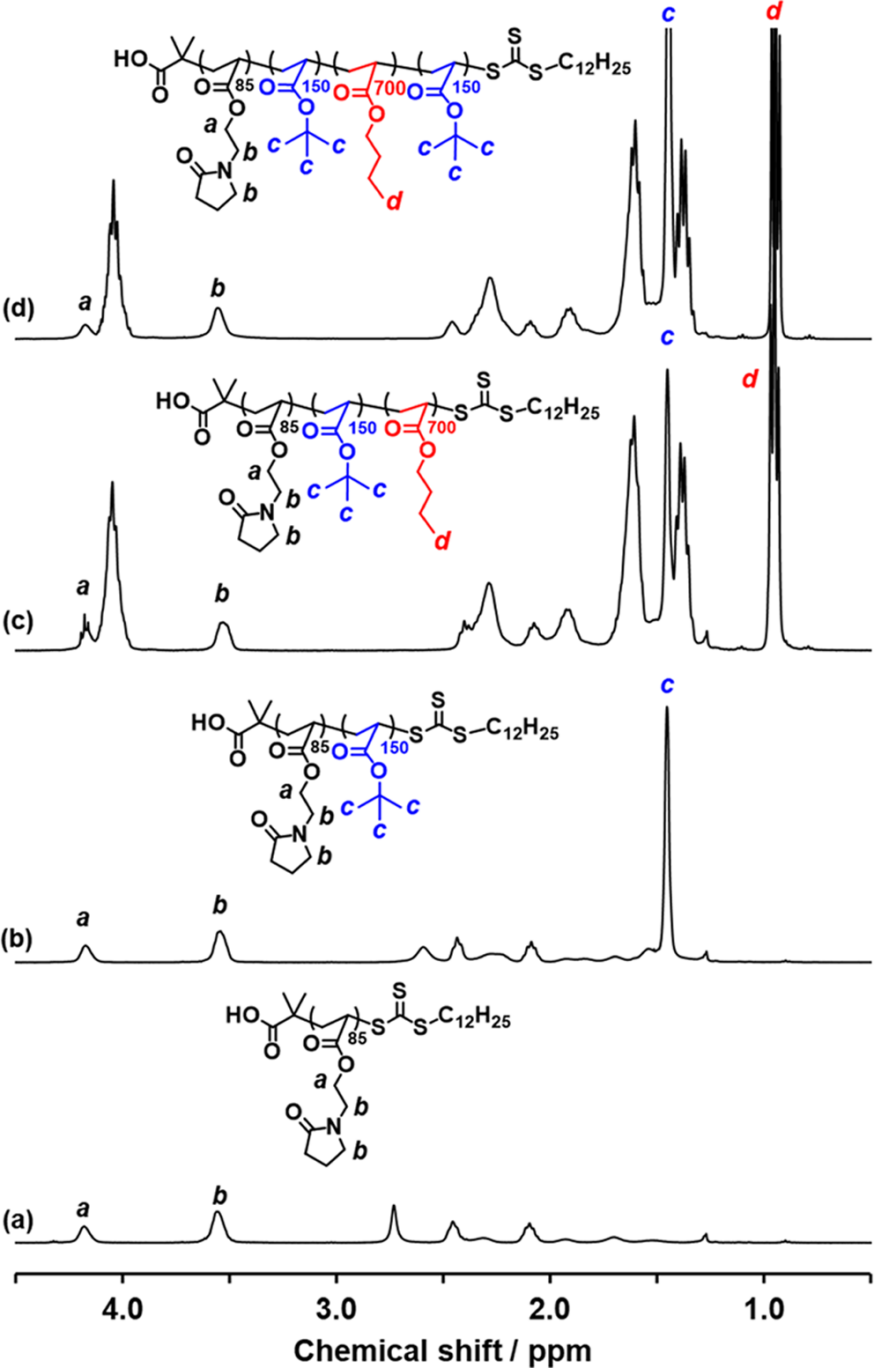
Partially assigned ^1^H NMR spectra (CDCl_3_) for each stage of the RAFT aqueous emulsion copolymerization synthesis outlined in Scheme 1: (a) PNAEP_85_ precursor, (b) initial PNAEP_85_-PtBA_150_ diblock copolymer, (c) intermediate PNAEP_85_-PtBA_150_-PnBA_700_ triblock copolymer, and (d) final PNAEP_85_-PtBA_150_-PnBA_700_-PtBA_150_ tetrablock copolymer. In each spectrum, the protons associated with the growing block do not overlap with the proton signals assigned to the PNAEP stabilizer block.

At this point, nBA and the KPS/AsAc initiator dissolved in dilute aqueous HCl (pH 3) were added to the reaction mixture under a nitrogen atmosphere. Although tBA and nBA are structural isomers, unique ^1^H NMR signals could be identified for the corresponding blocks, see Figure 3. More specifically, the instantaneous monomer conversion at the first stage of the emulsion polymerization was calculated from the attenuated tBA monomer vinyl signals relative to the tertiary methyl protons *c* assigned to PtBA. Subsequently, the attenuated nBA monomer vinyl signals were compared to the pendent methyl protons *d* assigned to PnBA. ^1^H NMR analysis indicated that 20% nBA conversion was achieved within 5 min at 30 °C. This stage was allowed to proceed for 40 min to ensure a high final nBA conversion (>95%; see red triangles in Figure 2) prior to the final stage. Attempts to increase the PnBA DP above 700 via RAFT emulsion polymerization at pH 3 resulted in incomplete conversion (<85%). A strategy to overcome this problem is discussed later.

To complete the tetrablock copolymer synthesis, tBA monomer plus further redox initiator (dissolved in dilute aqueous HCl; pH 3) were added after 70 min. No induction period was observed in this case, which suggests rapid diffusion of monomer into the nascent nanoparticles. Moreover, ^1^H NMR analysis confirmed that a high overall comonomer conversion was achieved within 40 min, as evidenced by the almost complete disappearance of all monomer vinyl signals (see purple squares in Figure 2). These kinetic studies indicate that PNAEP_85_-PtBA_150_-PnBA_700_-PtBA_150_ tetrablock copolymer nanoparticles can be prepared at up to 20% w/w solids within 2 h at 30 °C via RAFT aqueous emulsion polymerization using a convenient one-pot protocol. It is perhaps worth emphasizing that this overall time scale is much shorter than that required for various literature syntheses of multiblock copolymer nanoparticles, which typically require 3 to 18 h per block.^20−22^

**Figure 4:**
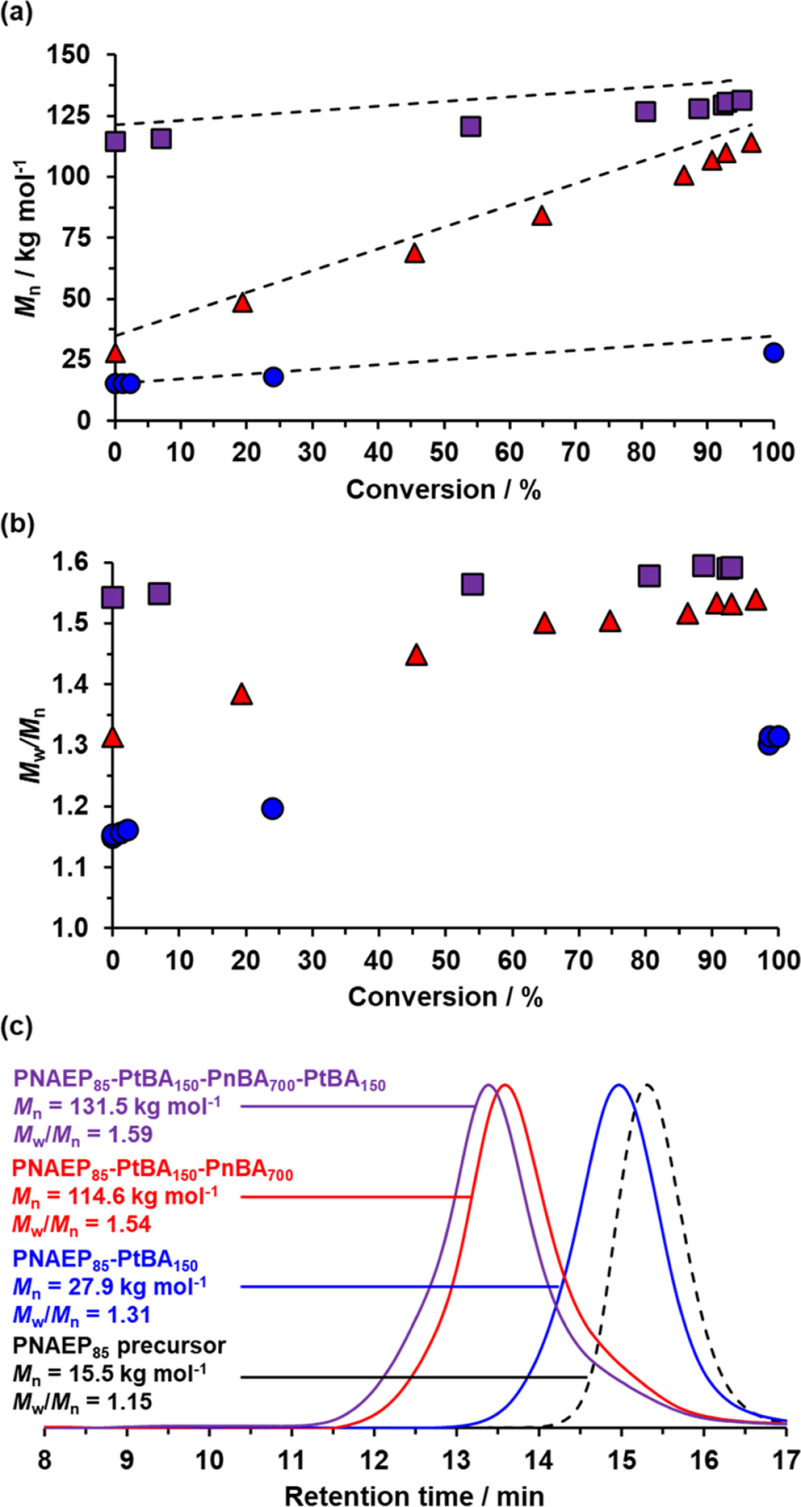
DMF GPC data illustrating the evolution in (a) *M*_n_ and (b) *M*_w_/*M*_n_ vs monomer conversion for growth of the first PtBA block (blue circles), the PnBA block (red triangles), and the second PtBA block (purple squares) during the synthesis of PNAEP_85_-PtBA_150_-PnBA_700_-PtBA_150_ tetrablock copolymer nanoparticles via one-pot RAFT aqueous emulsion copolymerization at 30 °C when targeting 20% w/w solids at pH 3. In part (a), the dashed lines represent the theoretical *M*_n_ values for each block. (c) GPC curves recorded during the synthesis of the PNAEP_85_-PtBA_150_-PnBA_700_-PtBA_150_ tetrablock copolymer chains after more than 95% monomer conversion had been observed for each individual block (see Figure 2). GPC data were obtained using a refractive index detector and are expressed relative to a series of near-monodisperse poly(methyl methacrylate) calibration standards.

Each of the aliquots extracted during the ^1^H NMR kinetic study was also analyzed by DMF GPC to monitor the evolution in the copolymer molecular weight distribution (see Figure 4). A linear increase in *M*_n_ with monomer conversion was observed during each stage of this synthesis (Figure 4a). As expected, a relatively large increase in molecular weight (86.7 kg mol^–1^) was observed during the synthesis of the PnBA_700_ block, whereas two rather smaller increases in molecular weight (12.4 and 16.9 kg mol^–1^, respectively) occurred during the synthesis of each PtBA_150_ block. The latter difference arises simply because PNAEP_85_ block contributes around 43% to the overall *M*_n_ observed for the initial PNAEP_85_-PnBA_150_ diblock copolymer but comprises only approximately 10% of the much higher *M*_n_ of the final tetrablock copolymer. Moreover, all such *M*_n_ data are apparent values that are expressed relative to a series of poly(methyl methacrylate) calibration standards. The copolymer molecular weight distribution gradually broadened during the polymerization (Figure 4b), with an *M*_w_/*M*_n_ of 1.59 being observed for the final tetrablock copolymer. This is significantly higher than that expected for a well-controlled RAFT polymerization, for which *M*_w_/*M*_n_ values below 1.50 are routinely reported.^75−77^ However, it compares quite favorably to GPC data reported in the literature for various multiblock copolymer nanoparticles prepared by RAFT aqueous emulsion polymerization.^21,22,52^ Moreover, GPC curves obtained using either a refractive index detector (solid purple line in Figure S2) or a UV detector (dashed purple line in Figure S2) were comparable, which suggests that the relatively broad molecular weight distribution obtained for the final tetrablock copolymer is simply the result of chain transfer to polymer. This is a well-documented side reaction for acrylic polymers, particularly when targeting higher-molecular-weight chains.^78−80^ It is perhaps worth emphasizing that the relatively high dispersity indicates that *M*_w_ is significantly greater than *M*_n_. This is likely to be beneficial in terms of the mechanical properties exhibited by the corresponding tetrablock copolymer films because the *M*_w_ is more likely to exceed *M*_e_ (see below). Finally, it is noteworthy that a relatively high blocking efficiency is obtained for each stage of this RAFT aqueous emulsion copolymerization (see Figure 4c). Furthermore, GPC studies indicated that the *M*_n_ increased linearly with the target PnBA DP (see Table 1).

**Table 1:** Summary of GPC and DLS Data Obtained during the Synthesis of Three Examples of PNAEP_85_-PtBA_150_-PnBA_*x*_-PtBA_150_ Tetrablock Copolymer Nanoparticles (Where *x* = 200, 400 or 700) Prepared Using a One-Pot RAFT Aqueous Emulsion Copolymerization Protocol at 30 °C When Targeting 20% w/w Solids at pH 3

	DMF GPC^a^		DLS	
polymer composition	*M*_n_, kg mol^–1^	*M*_w_/*M*_n_	*Z*-average diameter, nm	polydispersity
PNAEP_85_-PtBA_150_	26.3	1.36	66	0.11
PNAEP_85_-PtBA_150_-PnBA_200_	57.5	1.46	89	0.10
PNAEP_85_-PtBA_150_-PnBA_200_-PtBA_150_	70.4	1.58	101	0.07
PNAEP_85_-PtBA_150_	26.2	1.34	66	0.08
PNAEP_85_-PtBA_150_-PnBA_400_	76.1	1.48	102	0.09
PNAEP_85_-PtBA_150_-PnBA_400_-PtBA_150_	92.5	1.60	118	0.11
PNAEP_85_-PtBA_150_	27.9	1.31	68	0.09
PNAEP_85_-PtBA_150_-PnBA_700_	114.6	1.54	123	0.11
PNAEP_85_-PtBA_150_-PnBA_700_-PtBA_150_	131.5	1.59	138	0.11

a Refractive index detector, DMF eluent, PMMA calibration standards.

**Figure 5:**
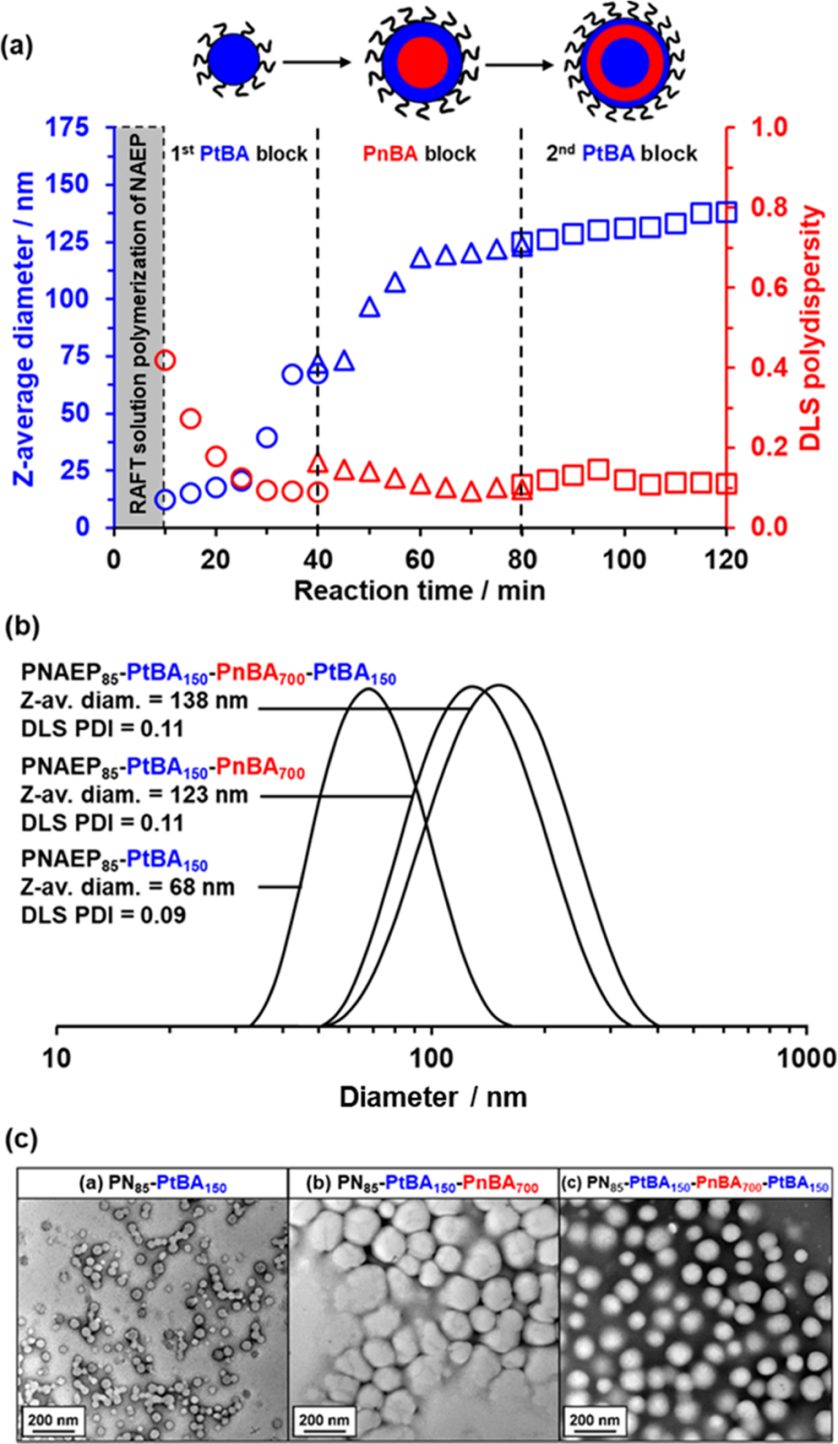
(a) Evolution of *z*-average diameter and polydispersity as determined by DLS studies during the synthesis of PNAEP_85_-PtBA_150_-PnBA_700_-PtBA_150_ tetrablock copolymer nanoparticles via RAFT aqueous emulsion copolymerization at 30 °C when targeting 20% w/w solids at pH 3. Vertical dashed lines indicate the time point at which each comonomer was added to the reaction mixture. (b) DLS particle size distributions recorded for the diblock, triblock, and tetrablock copolymer nanoparticles formed after each stage of this RAFT aqueous emulsion copolymerization (>95% monomer conversion was achieved in each case). (c) Corresponding TEM images recorded for (i) initial PNAEP_85_-PtBA_150_ spheres, (ii) intermediate PNAEP_85_-PtBA_150_-PnBA_700_ spheres, and (iii) final PNAEP_85_-PtBA_150_-PnBA_700_-PtBA_150_ spheres.

At first sight, the isomeric nature of PtBA and PnBA might be expected to lead to only weak (or perhaps no) segregation between these two hydrophobic blocks in the solid state.^37^ However, the *T*_g_ values for PnBA homopolymer (−54 °C)^61−63^ and PtBA (29–50 °C)^60^ differ significantly, which should promote microphase separation. According to Zetterlund and co-workers, hydrophobic RAFT Z groups (such as that conferred by DDMAT) should be located within the growing nanoparticle cores.^22^ In principle, this should lead to the formation of multilayered onion-like nanoparticles (see Scheme 1 and Figure 5a). However, it is also conceivable that the central PnBA block could displace the PtBA chains from the copolymer/water interface.^81^ A prerequisite for this scenario is that the reaction temperature should be above the *T*_g_ of the PnBA block to ensure sufficient chain mobility. Since these copolymer syntheses are performed at 30 °C, such displacement may be feasible.

DLS studies of aliquots extracted from the reaction mixture during the RAFT aqueous emulsion polymerization of tBA confirmed the formation of well-defined nanoparticles, with a significant increase in the scattered light intensity (or derived count rate), indicating that micellar nucleation occurred within 25 min (Figure 5a). Well-defined PNAEP_85_-PtBA_150_ nanoparticles with a z-average diameter of 68 nm and a DLS polydispersity of 0.09 were formed within 40 min, with this time scale corresponding to the end of the tBA polymerization (see Figure 5b). TEM analysis revealed the presence of spherical nanoparticles with a number-average diameter of 43 ± 6 nm (see Figure 5c). The significant discrepancy between the DLS and TEM diameter occurs for two reasons. First, these two techniques measure different moments of the particle size distribution, so DLS will always oversize relative to TEM for any size distribution of finite width. Second, DLS reports the overall hydrodynamic diameter for these sterically stabilized nanoparticles, whereas TEM is only sensitive to the PtBA nanoparticle cores.

Subsequent addition of nBA monomer led to an increase in the z-average diameter and DLS polydispersity within 5 min, which is ascribed to the formation of monomer-swollen nanoparticles (Figure 5a). This is consistent with the fact that no induction period was observed for this second-stage polymerization (see Figure 2). A gradual increase in z-average diameter and reduction in DLS polydispersity were observed during the subsequent nBA polymerization, resulting in the formation of relatively large, well-defined PNAEP_85_-PtBA_150_-PnBA_700_ triblock copolymer nanoparticles (final z-average diameter = 123 nm, DLS polydispersity = 0.11; see Figure 5a,b). A close inspection of the TEM image shown in Figure 5c indicates the presence of partially fused aggregates (apparent number-average diameter = 141 ± 18 nm) comprising multiple spherical nanoparticles (apparent number-average diameter = 88 ± 21 nm). This artifact is simply the result of the relatively low *T*_g_ of the PnBA block, which comprises 72% of the triblock copolymer chains by mass. This leads to significant nanoparticle deformation during TEM grid preparation. Nevertheless, the presence of the high *T*_g_ PtBA component enabled useful TEM studies of these relatively soft nanoparticles. Only a modest increase in *z*-average diameter and DLS polydispersity was observed after the synthesis of the second PtBA block (Figure 5a). However, this was not unexpected, bearing in mind the relatively small DP difference between the intermediate triblock copolymer and the final tetrablock copolymer. After 120 min, the final PNAEP_85_-PtBA_150_-PnBA_700_-PtBA_150_ nanoparticles exhibited a z-average diameter of 138 nm and a DLS polydispersity = 0.11 (see Figure 5a,5b). As expected, the increase in PtBA content minimized film formation during TEM grid preparation.

TEM analysis confirmed a kinetically trapped spherical morphology (Figure 5c), which is often observed for RAFT aqueous emulsion polymerization formulations.^21,50,82^ Digital image analysis using ImageJ software indicated a number-average diameter of 102 ± 10 nm, which is consistent with the DLS data. DLS experiments confirmed the expected monotonic increase in the final z-average diameter. It is perhaps worth emphasizing that the initial PNAEP_85_-PtBA_150_ diblock copolymer nanoparticles/chains obtained for these three syntheses exhibited remarkably similar GPC and DLS data, which indicates rather good reproducibility for this one-pot aqueous PISA protocol. Unfortunately, all attempts to target PnBA DPs above 800 invariably resulted in substantially incomplete comonomer conversions, despite increasing the initiator concentration and extending the reaction time allowed for each block. Nevertheless, it is worth emphasizing that targeting an overall DP of more than 1000 for the PNAEP_85_-PtBA_700_-PnBA_700_-PtBA_150_ tetrablock copolymer requires a significantly lower concentration of DDMAT RAFT agent, which minimizes the color, cost, and malodor for such PISA formulations.

### SAXS Analysis

**Figure 6:**
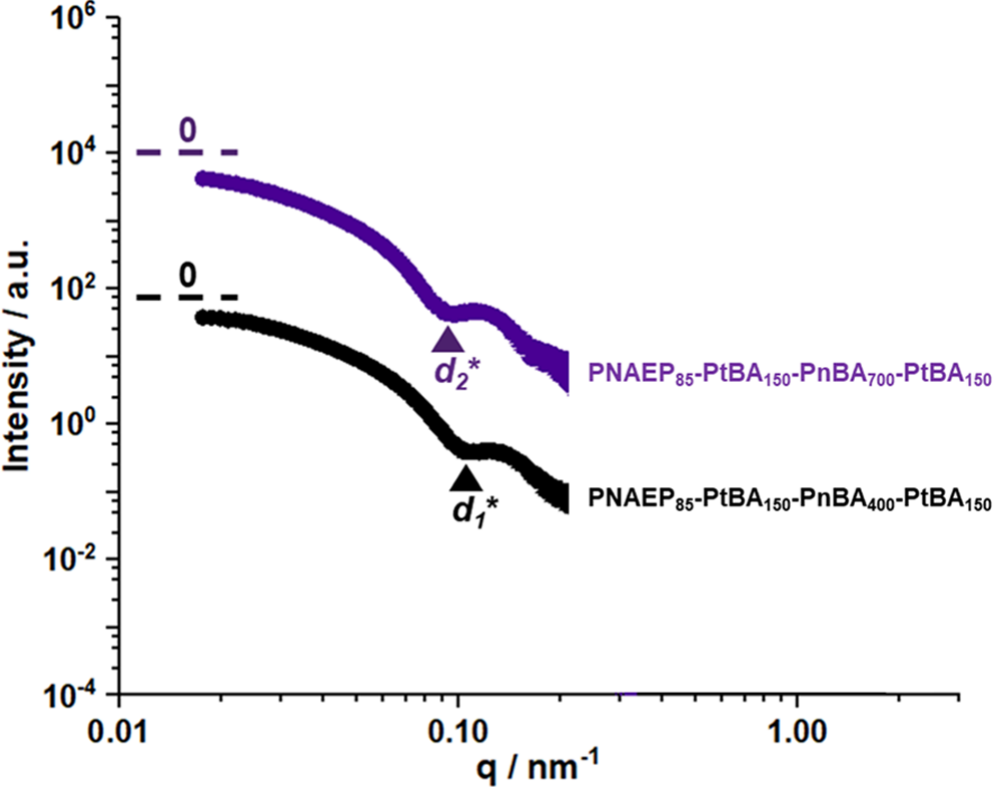
SAXS patterns recorded for 1.0% w/w aqueous dispersions of PNAEP_85_-PtBA_150_-PnBA_400_-PtBA_150_ and PNAEP_85_-PtBA_150_-PnBA_700_-PtBA_150_ tetrablock copolymer nanoparticles prepared by one-pot RAFT aqueous emulsion copolymerization. The dashed lines at low *q* represent zero gradients. The volume-average nanoparticle radius was estimated from the position of the first minimum (*q* = 0.0987 nm^–1^ for PnBA DP = 400 and *q* = 0.0863 nm^–1^ for PnBA DP = 700).

SAXS studies were conducted on 1.0% w/w aqueous dispersions of PNAEP_85_-PtBA_150_-PnBA_400_-PtBA_150_ and PNAEP_85_-PtBA_150_-PnBA_700_-PtBA_150_ tetrablock copolymer nanoparticles (Figure 6). It is well known that the low *q* gradient in an *I*(*q*) vs *q* plot (where *I*(*q*) is the scattering intensity and *q* is the scattering vector) is characteristic of the predominant copolymer morphology.^36,83,84^ For both SAXS patterns shown in Figure 6, this gradient tends toward zero, which is consistent with the presence of spherical nanoparticles.^85,86^ Moreover, the position of the first minimum (see *d*_1_* and *d*_2_* labels in Figure 6) can be used to estimate the nanoparticle core radius (*r*) using the well-known relation *r* = 4.49/*q*.^87^ This analysis indicated that the core diameter (or 2*r*) increased from 92 to 104 nm when the target PnBA DP was raised from 400 to 700.

This is consistent with the increase in *z*-average diameter from 118 to 138 nm indicated by DLS studies (see Table 1). However, the two SAXS patterns shown in Figure 5 could not be satisfactorily fitted using a well-known spherical micelle model,^88^ with significant deviations between the model fit and the experimental data being observed in the low *q* region. This discrepancy is likely to be related to the anticipated onion-like internal structure of these nanoparticles.

### Preparation and Characterization of PNAEP_85_-PtBA_150_-PnBA_*x*_-PtBA_150_ Tetrablock Copolymer Thermoplastic Elastomeric Films

**Figure 7:**
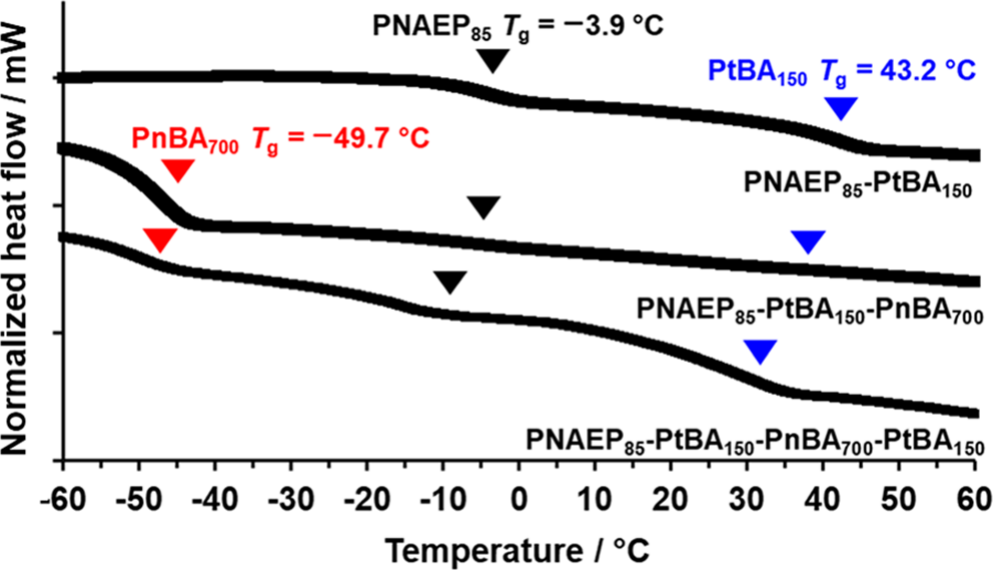
DSC curves recorded at a heating rate of 10 °C min^–1^ for PNAEP_85_-PtBA_150_ (top), PNAEP_85_-PtBA_150_-PnBA_700_ (middle) and PNAEP_85_-PtBA_150_-PnBA_700_-PtBA_150_ (bottom). Inverted triangles indicate the *T*_g_ values for PNAEP (black), PtBA (blue), and PnBA (red) homopolymers. [N.B. These curves are arbitrarily offset for the sake of clarity].

DSC was used to determine *T*_g_ values for three block copolymers: PNAEP_85_-PtBA_150_, PNAEP_85_-PtBA_150_-PnBA_700_, and PNAEP_85_-PtBA_150_-PnBA_700_-PtBA_150_ (Figure 7). PNAEP_85_-PtBA_150_ exhibited two distinct *T*_g_ values, indicating microphase separation between the hydrophilic and hydrophobic blocks. The *T*_g_ observed at −3.9 °C was assigned to the PNAEP_85_ precursor.^56,57^ The *T*_g_ at 43.2 °C was attributed to the PtBA_150_ block and is within the range reported for PtBA homopolymer in the literature (*T*_g_ = 29–50 °C).^60^ The DSC curve recorded for PNAEP_85_-PtBA_150_-PnBA_700_ indicated three distinct *T*_g_ values. The prominent *T*_g_ at −46.8 °C is close to the literature value for PnBA homopolymer and reflects the relatively high mass fraction (72%) for this component.^61−63^ Two weaker features corresponding to *T*_g_ transitions for the PNAEP_85_ and PtBA_150_ blocks were also discernible. The *T*_g_ observed for the PtBA block was reduced from 43.2 to 39.4 °C, which most likely reflects the fact that it is attached to the more mobile PnBA block.^43,44^ Three distinct *T*_g_ values were observed for PNAEP_85_-PtBA_150_-PnBA_700_-PtBA_150_ owing to its higher PtBA content. However, the PtBA *T*_g_ was further lowered to 34.2 °C [N.B. ^1^H NMR spectroscopy studies of these four copolymers indicated no residual monomer]. The soft PnBA block and the hard PtBA block are structural isomers, so their enthalpic incompatibility is likely to be quite small. Stronger microphase separation is anticipated when at least one block has a relatively high molecular weight, which is the case for the PnBA block reported herein. Moreover, the difference in *T*_g_ between the PnBA and PtBA blocks is 93 °C, which may also play an important role in driving microphase separation to produce thermoplastic elastomers.

PNAEP_85_-PtBA_150_-PnBA_200_-PtBA_150_, PNAEP_85_-PtBA_150_-PnBA_400_-PtBA_150_, and PNAEP_85_-PtBA_150_-PnBA_700_-PtBA_150_ copolymer films were prepared by drying the respective 20% w/w aqueous dispersions at 20 °C for 24 h. The mean film thickness was varied between 50 and 200 μm by drying larger amounts of the 40% w/w dispersion (e.g., 1–5 g). A 150 μm PNAEP_85_-PtBA_150_-PnBA_200_-PtBA_150_ film displayed no thermoplastic elastomer behavior owing to its PtBA-rich content and hence was not studied further. Similar observations were made by Zhu and co-workers for PS-rich PS-PnBA-PS triblock copolymer films.^20^ Digital photographs of the copolymer films are shown in Figure S3. The PNAEP_85_-PtBA_150_-PnBA_400_-PtBA_150_ film was significantly more colored owing to its lower overall DP and therefore higher concentration of trithiocarbonate end groups, while the PNAEP_85_-PtBA_150_-PnBA_700_-PtBA_150_ film was more transparent. Preliminary tensile tests were performed by simply hand-stretching these films, with digital photographs being recorded in their original relaxed state (see Figure S3a,c) and at their maximum elongation just prior to film rupture (see Figure S3b,d). On release of the stretched films, they regained their original dimensions (see Figure S3e). Moreover, increasing the content of the low-*T*_g_ block produces more elastic films.

**Figure 8:**
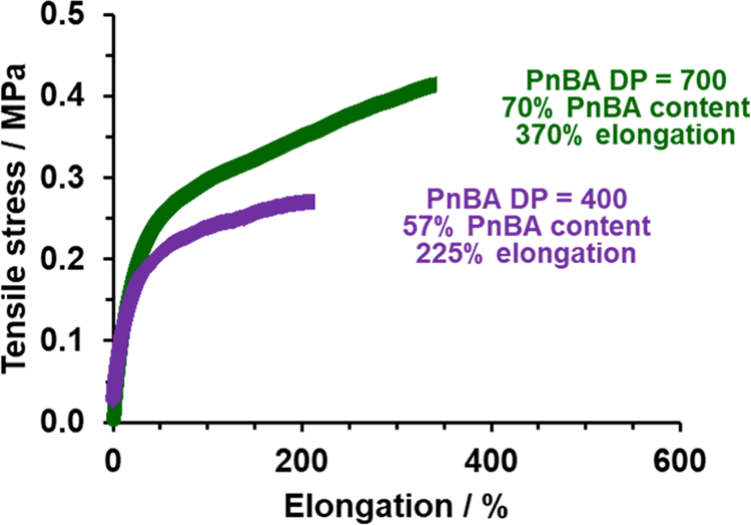
Stress–strain curves recorded for PNAEP_85_-PtBA_150_-PnBA_*x*_-PtBA_150_ [where *x* = 400 (purple curve) or 700 (green curve)] tetrablock thermoplastic elastomeric films prepared by drying 20% w/w aqueous copolymer dispersions at ambient temperature. PnBA contents are expressed in mass %.

The stress–strain behavior of a pair of PNAEP_85_-PtBA_150_-PnBA_*x*_-PtBA_150_ (where *x* = 400 or 700) tetrablock thermoplastic elastomer films was assessed via uniaxial tensile measurements (see Figure 8). Increasing the DP of the low-*T*_g_ PnBA block from 400 to 700 produced a significantly stronger, more elastic film (maximum strength >0.4 MPa; elongation at break = 370%). Such mechanical data are comparable to that reported for commercial all-acrylic thermoplastic elastomers in the literature.^89^ Adjusting the target PnBA DP from 400 to 700 increased the Young’s modulus by 25% and film toughness by 120% (see Table S1). Moreover, targeting a longer PnBA block also reduces the organosulfur content of the copolymer film, with a concomitant reduction in both color (see Figure S4) and malodor. Given that RAFT agents are relatively expensive compared to the other components of such formulations, this approach also lowers the overall manufacturing cost.

**Figure 9:**
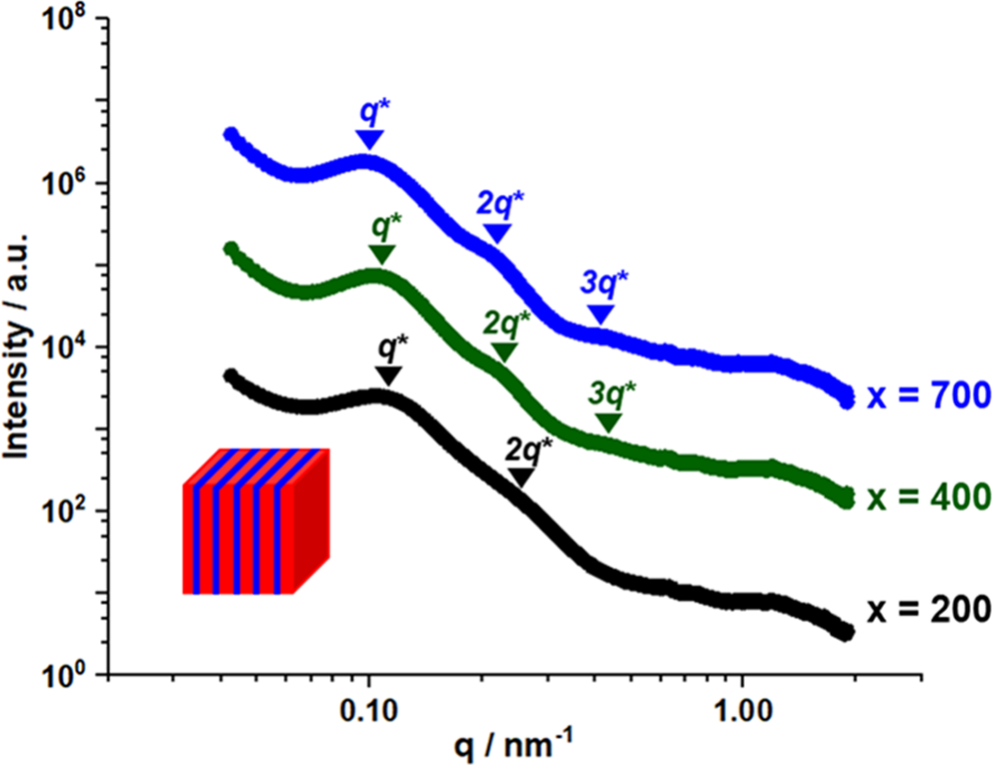
SAXS patterns recorded for PNAEP_85_-PtBA_150_-PnBA_200_-PtBA_150_ (black), PNAEP_85_-PtBA_150_-PnBA_400_-PtBA_150_ (green), and PNAEP_85_-PtBA_150_-PnBA_700_-PtBA_150_ (blue) tetrablock copolymer films dried at 20 °C without annealing. Higher-order scattering peaks are labeled relative to the principal scattering peaks (q*). The inset schematic cartoon shows the lamellar phase morphology for each of these three tetrablock copolymers (red = PnBA and blue = PtBA), as suggested by such SAXS data.

SAXS was used to characterize the extent of microphase separation within PNAEP_85_-PtBA_150_-PnBA_200_-PtBA_150_ (black trace in Figure 9), PNAEP_85_-PtBA_150_-PnBA_400_-PtBA_150_ (green trace in Figure 9), and PNAEP_85_-PtBA_150_-PnBA_700_-PtBA_150_ (blue trace in Figure 9) tetrablock copolymer films obtained after drying at 20 °C for 24 h. In each case, a relatively broad principal scattering peak was identified (see Bragg reflections labeled q*). This feature can be used to calculate the mean domain size *d* within the microphase-separated films using the well-known relation *d* = 2π/q*.^87,90^ Analysis indicated that *d* increased from 57 to 64 Å when increasing the PnBA DP from 200 to 700.

The SAXS patterns recorded for the PNAEP_85_-PtBA_150_-PnBA_400_-PtBA_150_ and PNAEP_85_-PtBA_150_-PnBA_700_-PtBA_150_ films both exhibit three higher-order structure peaks at q*, 2q* and 3q* (Figure 9), which is consistent with a lamellar structure.^90^ In contrast, the PNAEP_85_-PtBA_150_-PnBA_200_-PtBA_150_ film has a very weak second-order peak and no discernible third-order peak. Its lack of long-range translational order is typically observed when χ is low and is consistent with the very poor elastomeric properties observed for this film.^27^

According to the literature, lamellar phases usually lead to film failure at relatively low elongation at break values.^30,91−94^ Superior thermoplastic elastomer performance is usually obtained when either spherical or cylindrical hard block domains are uniformly distributed within a continuous soft block matrix.^90,95^ This suggests that stronger, more resilient thermoplastic elastomers (compared to those shown in Figure S3) should be accessible if such copolymer morphologies can be produced. This clearly warrants further examination for such aqueous PISA formulations. Finally, one of the reviewers of this manuscript has noted that the *T*_g_ of the “hard” PtBA block is relatively low for a thermoplastic elastomer, which may limit the service temperature of the tetrablock copolymer films reported herein. In principle, this problem could be addressed by replacing the tBA monomer with adamantyl acrylate.^89^ Unfortunately, this refinement is beyond the scope of the current study.

### Further Syntheses of PNAEP_85_-PtBA_150_-PnBA_*x*_-PtBA_150_ Tetrablock Copolymer Nanoparticles

As described above, using a low-temperature KPS/AsAc redox initiator enabled the synthesis of multiblock thermoplastic elastomers at pH 3. However, only relatively low PnBA DPs (<700) could be targeted at less than 25% w/w solids; attempts to increase either parameter invariably resulted in incomplete monomer conversion and loss of colloidal stability. Recently, Zetterlund and co-workers reported that optimization of RAFT emulsion polymerization formulations was required for successful multiblock copolymer syntheses.^53^ More specifically, they found that using more hydrophobic radicals led to greater RAFT control (lower *M*_w_/*M*_n_ values) when preparing nanoparticles comprising high *T*_g_ copolymer cores. In contrast, the hydrophobic character of the radical species had little influence on the RAFT control achieved during the synthesis of nanoparticles with low-*T*_g_ copolymer cores. Inspired by these observations, we evaluated an alternative low-temperature KPS/TMEDA redox initiator at pH 7 (rather than pH 3).^96^ Under such conditions, the carboxylic acid located at the end of each PNAEP stabilizer chain is ionized, which confers greater colloidal stability on the growing block copolymer nanoparticles.^97^ Moreover, the relatively low reaction temperature employed to prevent ester hydrolysis of the tBA and nBA comonomers and minimize chain transfer to the polymer can be maintained.^98^

**Figure 10:**
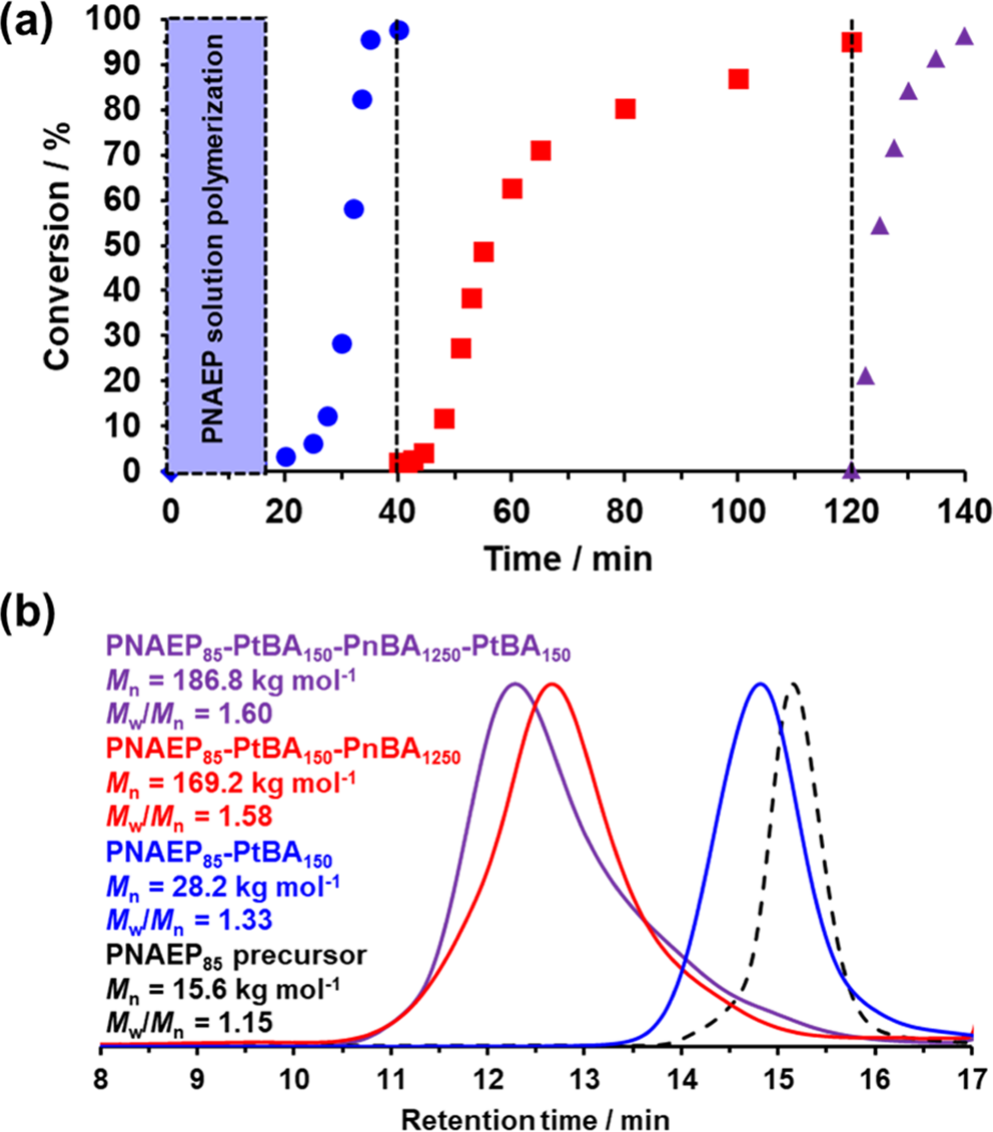
(a) Conversion vs time curves determined from ^1^H NMR spectroscopy studies during the synthesis of PNAEP_85_-PtBA_150_-PnBA_1250_-PtBA_150_ tetrablock copolymer nanoparticles at 40% solids, pH 7, and 30 °C via the one-pot sequential RAFT aqueous emulsion copolymerization of tBA (blue circles), nBA (red squares), and tBA (purple triangles) using a PNAEP_85_ precursor prepared. Vertical dashed lines indicate the injection times for the tBA, nBA, and tBA monomers during this aqueous PISA synthesis. (b) DMF GPC curves recorded during the synthesis of the PNAEP_85_-PtBA_150_-PnBA_1250_-PtBA_150_ tetrablock copolymer at pH 7 after more than 95% monomer conversion had been achieved for each individual block. GPC data are expressed relative to a series of near-monodisperse poly(methyl methacrylate) calibration standards.

The synthesis of PNAEP_85_-PtBA_150_-PnBA_1250_-PtBA_150_ tetrablock copolymers via RAFT aqueous emulsion polymerization was attempted using the KPS/TMEDA redox initiator pair at pH 7 and 30 °C (see Scheme S1). ^1^H NMR spectroscopy studies indicated that high monomer conversions (>95%) could be achieved at each stage of polymerization, and the tetrablock copolymer synthesis was complete within 140 min (see Figure 10a).

Surprisingly, the RAFT emulsion polymerization of nBA (target PnBA DP = 1250) using the PNAEP_85_-PtBA_150_ diblock copolymer precursor was complete within 60 min (Figure 10a). Figure 4a indicates that 40 min is required to achieve high nBA monomer conversion when targeting a PnBA DP of 750. Thus, only a modest increase in polymerization time (20 min) is required to achieve a 67% increase in target DP. It is worth emphasizing that all attempts to target PnBA DPs above 750 were unsuccessful when using a KPS/AsAc redox initiator at pH 3: macroscopic precipitation was invariably observed, and comonomer conversions remained substantially incomplete, even when employing long reaction times (>24 h). In contrast, using the KPS/TMEDA redox initiator at pH 7 produces an anionic carboxylate group at the end of each steric stabilizer chain, which confers enhanced colloidal stability via electrosteric stabilization.

Aliquots taken during the synthesis of the PNAEP_85_-PtBA_150_-PnBA_1250_-PtBA_150_ tetrablock copolymer that corresponded to complete monomer conversion (>95%) were analyzed using DMF GPC studies (Figure 10b). These studies indicated that the PNAEP_85_ homopolymer synthesis was well controlled and comparable to that conducted at pH 3 (15.6 kg mol^–1^; *M*_w_/*M*_n_ = 1.16; see Table 1 for pH 3 comparison). The synthesis of the PNAEP_85_-PtBA_150_ diblock copolymer was also reasonably well controlled, as judged by the relatively narrow molecular weight distribution. A somewhat broader molecular weight distribution was observed for the PNAEP_85_-PtBA_150_-PnBA_1250_ triblock copolymer, but such data compare quite favorably with that reported in the literature for the synthesis of high molecular weight triblock copolymers via RAFT polymerization.^19−21^ Finally, the GPC data recorded during the synthesis of PNAEP_85_-PtBA_150_-PnBA_1250_-PtBA_150_ tetrablock nanoparticles at pH 7 indicated that reasonably high chain extension efficiencies were achieved for each block, and the molecular weight increased proportionately in each case.

## Conclusions

Highly transparent waterborne thermoplastic elastomers based on two structural isomers, *t*-butyl acrylate and *n*-butyl acrylate, were prepared by a convenient one-pot protocol involving RAFT aqueous emulsion copolymerization. This highly efficient, low-viscosity formulation enabled PNAEP_85_-PtBA_150_-PnBA_700_-PtBA_150_ tetrablock copolymer nanoparticles to be prepared at 20% w/w solids within 2 h at 30 °C using a low-temperature redox initiator.

Three examples of PNAEP_85_-PtBA_150_-PnBA_*x*_-PtBA_150_ tetrablock copolymer nanoparticles (where *x* = 200, 400, or 700) were analyzed by GPC, DLS, DSC, and SAXS. A linear increase in *M*_n_ with conversion of each block was observed, suggesting that living character was maintained throughout the polymerization. The nanoparticle diameter increased monotonically over time and correlated well with the respective comonomer conversion curves and target molecular weight for each block. DSC studies of PNAEP_85_-PtBA_150_, PNAEP_85_-PtBA_150_-PnBA_700_, and PNAEP_85_-PtBA_150_-PnBA_700_-PtBA_150_ indicated that the *T*_g_ difference between the hard and soft blocks was approximately 93 °C. The all-acrylic nature of such tetrablock copolymer dispersions enabled the formation of highly transparent films on drying at 20 °C. The PNAEP_85_-PtBA_150_-PnBA_200_-PtBA_150_ film displayed no elastomeric properties owing to the insufficiently short low-*T*_g_ PnBA block. In contrast, the elongation at break increased from 225% (PnBA DP = 400) to 375% (PnBA DP = 700) for the other two tetrablock copolymer films, which confirms that such materials behave as thermoplastic elastomers. SAXS was used to characterize the extent of phase separation within the three tetrablock copolymer films. Each film exhibited a lamellar structure, with greater micro-phase separation being observed for higher molecular weight copolymers. Literature precedent indicates that the preparation of tetrablock copolymer films comprising either spherical or cylindrical domains should lead to enhanced elastomeric properties.^90^

Finally, a KPS/TMEDA redox initiator was used to conduct the same RAFT emulsion polymerization syntheses at pH 7. This led to ionization of the carboxylic acid end-groups, which conferred electrosteric stabilization. This ensured that good colloidal stability was maintained for the tetrablock copolymer nanoparticles even at 40% w/w solids and also enabled a significantly higher DP of 1250 to be achieved for the low-*T*_g_ PnBA block. In summary, these new aqueous PISA formulations represent important progress regarding the rational design of waterborne thermoplastic elastomers based on film-forming multiblock copolymer nanoparticles.

## Supporting Information Available

Schematic synthesis of PNAEP_85_-PtBA_150_-PnBA_700_-PtBA_150_ tetrablock copolymer nanoparticles at 40% solids, pH 7, and 30 °C; GPC curves recorded for a PNAEP_85_-PtBA_150_-PnBA_700_-PtBA_150_ tetrablock copolymer; digital photographs recorded during preliminary tensile tests on tetrablock copolymer films; table containing linear regression data for Young’s modulus and area under the curve for toughness; digital photographs recorded showing the reduction in color at higher target DPs. (PDF)
