# Supplementary material for: Synthesis and Characterization of All-Acrylic Tetrablock Copolymer Nanoparticles: Waterborne Thermoplastic Elastomers via One-Pot RAFT Aqueous Emulsion Polymerization
Source: Chem Mater. 2024 Feb 14;36(4):2061–75. doi: 10.1021/acs.chemmater.3c03115 (PMC10902817; doi:10.1021/acs.chemmater.3c03115)
Supplement: Supplementary file 1 — cm3c03115_si_001.pdf [file cm3c03115_si_001.pdf]

Supporting Information for

Synthesis and Characterization of All-Acrylic Tetrablock Copolymer Nanoparticles: Waterborne Thermoplastic Elastomers via One-Pot RAFT Aqueous Emulsion Polymerization

Oliver J. Deane,[a] Pierre Mandrelier,[a] Osama M. Musa,[b] Mohammed Jamali,[c] Lee A. Fielding,[c] and Steven P. Armes[a,*]

a. Dainton Building, Department of Chemistry, University of Sheffield, Brook Hill, Sheffield, South Yorkshire, S3 7HF, UK.

b. Ashland Specialty Ingredients, 1005 US 202/206, Bridgewater, New Jersey, 08807, USA.

c. School of Materials, The University of Manchester, Oxford Rd, Manchester, M13 9PL, UK

Supporting Figures

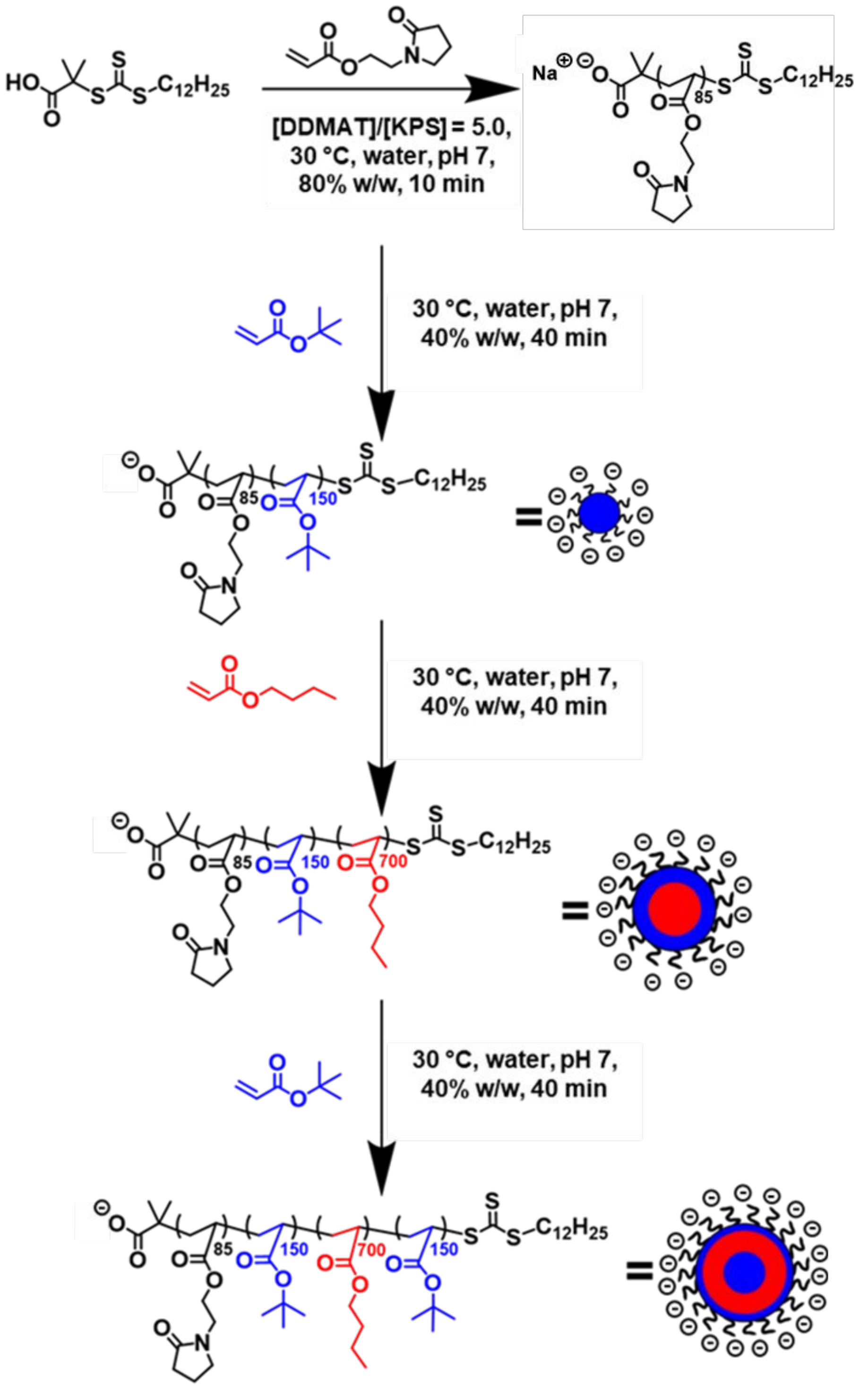

Scheme S1. Synthesis of PNAEP$_{85}$-PtBA$_{150}$-PnBA$_{700}$-PtBA$_{150}$ tetrablock copolymer nanoparticles at pH 7 via initial RAFT aqueous solution polymerization at 30 °C followed by three successive RAFT aqueous emulsion polymerizations conducted at the same temperature. N.B. This higher solution pH causes ionization of the terminal carboxylic acid groups.

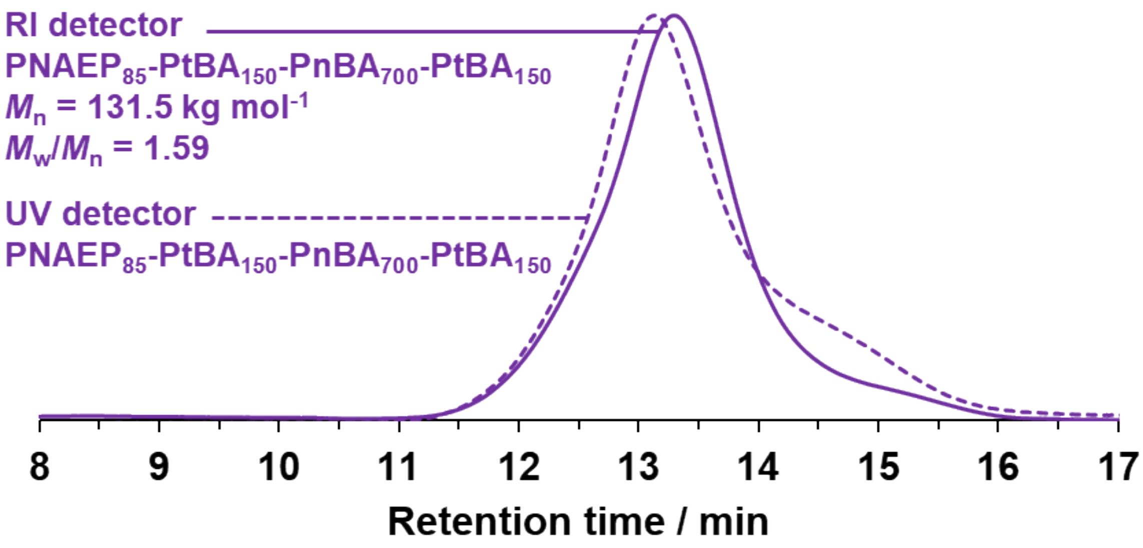

Figure S2. GPC curves recorded for a $PNAEP_{85}$-$PtBA_{150}$-$PnBA_{700}$-$PtBA_{150}$ tetrablock copolymer using either an RI detector (solid purple line) or a UV detector (dashed purple line). The apparent shift in retention time simply indicates that the UV detector is placed before the RI detector in the GPC set-up.

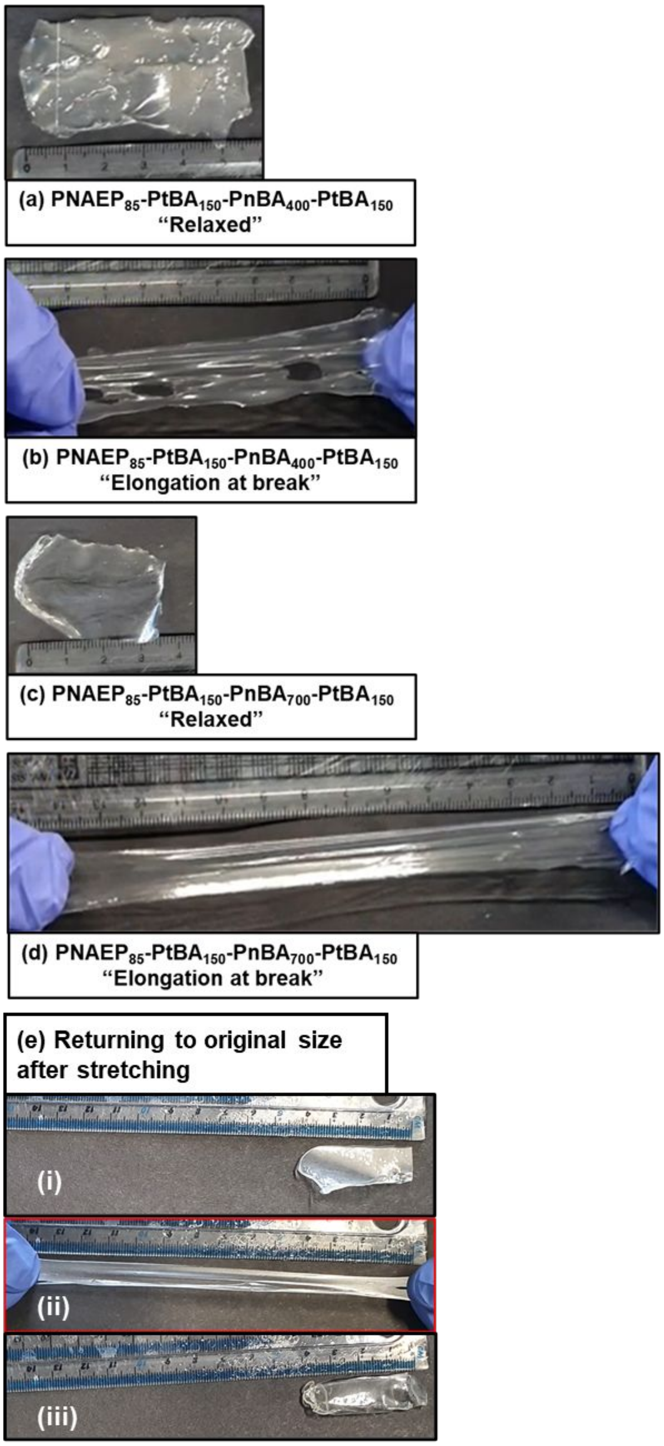

Figure S3. Digital photographs recorded during preliminary tensile tests on (a, b) $PNAEP_{85}$-$PtBA_{150}$-$PnBA_{400}$-$PtBA_{150}$ and (c, d) $PNAEP_{85}$-$PtBA_{150}$-$PnBA_{700}$-$PtBA_{150}$ films (mean film thickness = 150 ± 10 μm). (e) Images recorded (i) prior to stretching a $PNAEP_{85}$-$PtBA_{150}$-$PnBA_{700}$-$PtBA_{150}$ film (ii) the same film at its maximum elongation before break and (iii) the same film after returning to its original 4 cm length. These tetrablock copolymer films were prepared by drying the corresponding 40% w/w aqueous dispersions on Teflon sheets at 20 °C for 24 h.

Table S1. Summary of tensile test data for two $PNAEP_{85}$-$PtBA_{150}$-$PnBA_x$-$PtBA_{150}$ tetrablock copolymer films (x = 400 or 700) using linear regression for Young's modulus and determining the area under the curve for toughness.

PnBA DP (x)	Young's modulus[a] / MPa[a]	Toughness[b] / J m^{-3}
400	0.00415	56.18
700	0.00516	123.86

a. Calculated from the initial gradient.
b. Calculated from the area under the curve.

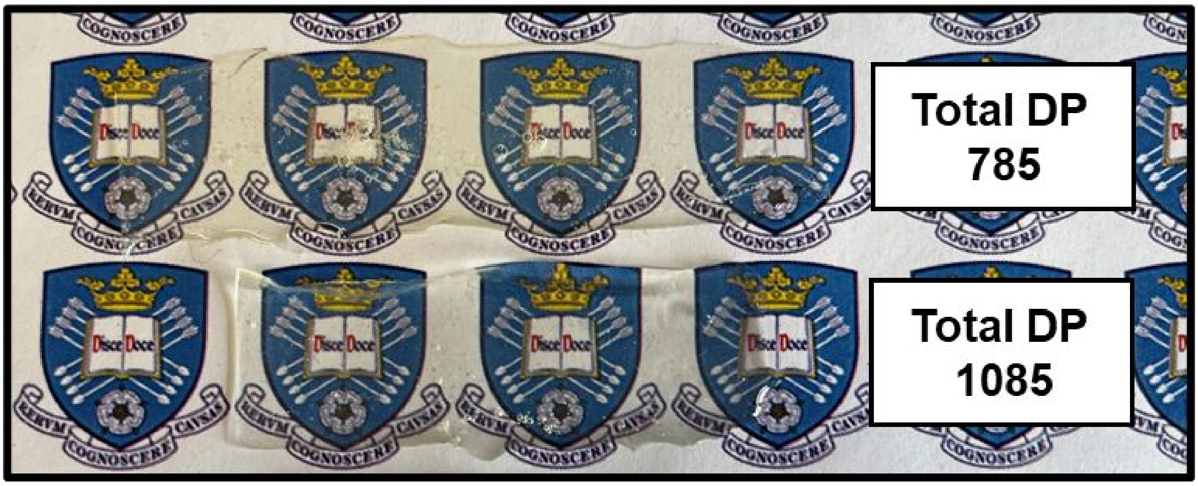

Figure S4. Digital photograph to illustrate the reduction in yellow coloration achieved when increasing the target x value for a $PNAEP_{85}$-$PtBA_{150}$-$PnBA_x$-$PtBA_{150}$ tetrablock copolymer film from 400 to 700 (which corresponds to increasing the overall target DP from 785 to 1085). Importantly, this change resulted in enhanced thermoplastic elastomer performance (see Figure 8 in the main manuscript).
